# Optimisation of Cu^+^ impregnation of MOF-74 to improve CO/N_2_ and CO/CO_2_ separations†

**DOI:** 10.1039/c9ra10115b

**Published:** 2020-01-31

**Authors:** Arwyn Evans, Matthew Cummings, Donato Decarolis, Diego Gianolio, Salman Shahid, Gareth Law, Martin Attfield, David Law, Camille Petit

**Affiliations:** Barrer Centre, Department of Chemical Engineering, Imperial College London UK camille.petit@imperial.ac.uk; School of Chemistry, The University of Manchester UK; Diamond Light Source Harwell Campus Didcot UK; BP Chemicals Ltd Petrochemicals Technology Saltend Hull UK

## Abstract

Carbon monoxide (CO) purification from syngas impurities is a highly energy and cost intensive process. Adsorption separation using metal–organic frameworks (MOFs) is being explored as an alternative technology for CO/nitrogen (N_2_) and CO/carbon dioxide (CO_2_) separation. Currently, MOFs' uptake and selectivity levels do not justify displacement of the current commercially available technologies. Herein, we have impregnated a leading MOF candidate for CO purification, *i.e.* M-MOF-74 (M = Co or Ni), with Cu^+^ sites. Cu^+^ allows strong π-complexation from the 3d electrons with CO, potentially enhancing the separation performance. We have optimised the Cu loading procedure and confirmed the presence of the Cu^+^ sites using X-ray absorption fine structure analysis (XAFS). *In situ* XAFS and diffuse reflectance infrared Fourier Transform spectroscopy analyses have demonstrated Cu^+^–CO binding. The dynamic breakthrough measurements showed an improvement in CO/N_2_ and CO/CO_2_ separations upon Cu impregnation. This is because Cu sites do not block the MOF metal sites but rather increase the number of sites available for interactions with CO, and decrease the surface area/porosity available for adsorption of the lighter component.

## Abstract 2

We present an *in situ* study of CO adsorption on Cu impregnated MOF-74 and study the competitive adsorption of CO *vs.* CO_2_ and N_2_.

## Graphical abstract

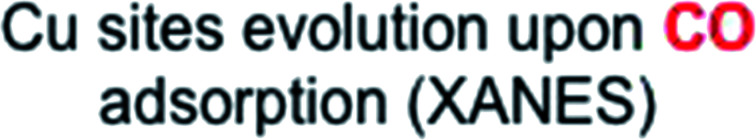

## 1. Introduction

The production of carbon monoxide (CO) as an industrial reagent requires downstream separation processes to achieve the high purity level required for CO to be used as a chemical feedstock (*i.e.* >99 mol%).^1,2^ During the CO purification process, by-product impurities such as CH_4_, CO_2_ and N_2_ must be removed. While current CO capture and gas separation technologies exist for purification,^2,3^ numerous energy-intensive steps are required which contribute to large capital and operating costs.^4^ The design of a single adsorption process to purify CO from all impurities would bring energy and cost savings.

Activated carbons (AC), zeolites and metal–organic frameworks (MOFs) have been identified for CO purification.^5^ Yet, their adsorption performance, especially ACs' and zeolites', fall below the CO capacity and selectivity levels required to justify the displacement of the current benchmark technology, *i.e.* cryogenic distillation.^2^ MOFs have shown the most promising performance to date,^6^ recording the highest CO adsorption uptakes and theoretical CO/X selectivities (where X = H_2_, N_2_ and CH_4_).^5,7^

MOFs are formed by organic linkers coordinating to metal nodes (or polynuclear clusters).^8^ Their metal sites can provide coordinatively unsaturated metal sites, also known as open-metal sites, which have an affinity for CO.^5^ Hence, the best performing MOFs are highly dependent on the MOFs' metal sites as they determine the metal–CO bond strength through both σ-bond formation and π-backdonation.^9^ A comprehensive study by Bloch *et al.* reported high CO capacities and CO/H_2_ and CO/N_2_ selectivities for MOF-74 (also known as CPO-27 or M_2_(DOBDC), DOBDC = 2,5-dioxido-1,4-benzenedicarboxylate).^7^ The study highlighted trends between the Irving–Williams series and the M^2+^ centres' affinity to CO, demonstrating the importance of the metal ion's electronic configuration upon CO separation.

To further improve MOFs' CO uptake, one can use metal impregnation, a method tested on ACs and zeolites.^9–18^ This approach increases selectivity by decreasing the uptake of the undesired light product (*e.g.* N_2_) whose affinity with the impregnated metal is low. The introduction of Cu^+^ metal sites is the most common example used for ACs and zeolites. The electronic configuration of Cu^+^ allows strong π-complexation from the 3d electrons.^9^ Several groups have produced Cu^+^-impregnated zeolites and activated carbons either through the use of a Cu^+^-containing salt or that of a Cu^2+^-containing salt with subsequent reduction.^9–18^ A similar route has also been conducted on Na–zeolite Y, initially through metal cation exchange with Cu^2+^ ions.^19,20^ These studies demonstrate that it is favourable to initially perform Cu^2+^ impregnation or ion-exchange, before reducing to Cu^+^. The Cu^+^ ions are prone to oxidation and reduction and therefore cannot be employed for direct ion-exchange or impregnation. While Cu impregnated materials typically perform better for CO separation than the unimpregnated materials, their CO capacity and theoretical selectivities still fall short of the best performing MOFs.^5,7^ Impregnation has also been performed on MOFs to improve their CO separation performance. Cu^+^ metal sites have been incorporated on Fe-MIL-100 for CO/N_2_*via* impregnation with Cu^2+^-based salts followed by reduction to Cu^+^.^21^ The same method has also been employed on Fe-MIL-100 for CO/CO_2_ separation,^22–24^ and on MIL-101 for CO/N_2_ separation.^25^ The CuAlCl_4_ complex used in the COSORB process^3^ has been loaded onto Cr-MIL-101 to improve CO/N_2_ separation and Cu^+^ stability. HKUST-1 has been used as a support for CuCl to improve CO/N_2_ and CO/H_2_ selectivity.^26^ A table comparing the performance of all these Cu impregnated MOFs is provided in the ESI (Table S1†).

Despite improving the CO purification ability, the CO capacity of Cu impregnated MOFs remains lower than those of most M-MOF-74 structures.^7^ Interestingly, Cu impregnation of MOF-74 has never been reported. Provided the new Cu sites do not block the MOF metal sites, this approach would increase the number of sites available for interactions with CO, while decreasing the surface area/pore size available for adsorption of the lighter component, *i.e.* N_2_. The combination of such effects should enhance CO uptake while increasing selectivity. Cu impregnation, particularly Cu^+^ impregnation, is notably challenging due to the unstable nature of the metal species. Monitoring the state of the Cu sites becomes important to understand the adsorption mechanism. To date, there has been no *in situ* analysis of the Cu^2+^ to Cu^+^ reduction procedure, and there has been no confirmation of Cu^+^–CO binding. X-ray photoelectron spectroscopy has been the sole characterisation technique used to confirm the presence of Cu^+^ by the absence of Cu^2+^ satellite peaks.^16,21^ Yet, this approach can only evaluate the surface, and not the bulk, chemistry of the Cu-impregnated MOFs. Furthermore, the absence of Cu^2+^ satellite peaks may be caused by Cu^2+^ to Cu^0^ over reduction rather than the formation of Cu^+^.

The present study aims to improve on the performance of MOF-74 for CO purification while bringing fundamental insight into Cu^+^ impregnation and Cu^+^–CO interactions in MOFs. To achieve this, we have performed a systematic Cu impregnation study on Ni-MOF-74 and Co-MOF-74.^5,7^ We have: (i) synthesized Cu^+^-impregnated MOF-74 structures to simultaneously use the metal–CO binding strength of the framework M^2+^ and introduce additional Cu^+^ ions, (ii) optimised the Cu impregnation and monitored *in situ* the Cu^2+^ to Cu^+^ reduction process using X-ray absorption near edge structure (XANES) analysis, (iii) verified Cu^+^–CO and M^2+^–CO binding using *in situ* DRIFTS analysis and (iv) tested Cu@Ni-MOF-74 for dynamic CO/N_2_ and CO/CO_2_ separation.

## 2. Experimental section

### 2.1 Materials synthesis

#### 2.1.1 MOF synthesis

Ni-MOF-74 and Co-MOF-74 were synthesised as described in the ESI, Section 1.2.†

#### 2.1.2 Cu impregnation procedure

M-MOF-74 (M = Ni or Co) was subjected to Cu impregnation. The procedure, described below, depended on the Cu salts used and the desired Cu impregnation level chosen.

For 200 mg of M-MOF-74, a total of 1.6 mL of Cu solution was used for this step and was kept constant for each synthesis. Aqueous solutions of the Cu salts were prepared by dissolving the salts in MeOH (for CuCl_2_) or MeCN (for CuCl and Cu(HCO_2_)_2_) through sonication. The Cu concentration depended on the Cu loading desired using 1.6 mL Cu solution. For Cu impregnation procedures with two salts (*e.g.* CuCl_2_ and Cu(HCO_2_)_2_), an equimolar solution was prepared.

The Cu solution and M-MOF-74 were sonicated in a 20 mL scintillation vial for 1 h, with the vial shaken by hand every 15 min to mix the suspension further. After sonication and allowing the suspension to settle, the excess solution was decanted and the remaining slurry was dried with flowing N_2_ overnight. The solvent removal for MOF activation and reduction of the Cu^2+^ to Cu^+^ sites are referred to as separate procedures throughout (activation and reduction), with the conditions varying depending on the procedure desired. The Cu impregnated M-MOF-74 structures are labelled *X*-Cu@M-MOF-74, where M = Ni, Co, and *X* represents the ratio of Cu over Ni in the samples, rounded to the nearest integer.

### 2.2 Materials characterisation

#### 2.2.1 Textural parameters

N_2_ adsorption and desorption isotherms were measured using a porosity analyser (Micromeritics 3Flex) at −196 °C. Prior to analysis, the samples were degassed by heating the samples at ∼0.2 mbar for 16 h at 120 °C to remove the majority of the residual solvent, and degassed *in situ* on the porosity analyser at ∼0.003 mbar for 6 h at 250 °C. The surface area was calculated using the Brunauer–Emmett–Teller (BET) method.^27^ The 3Flex software applied the Rouquerol criteria^28^ which can be used for MOFs.^29^ The total pore volume was calculated from the N_2_ adsorption at relative pressure (*P*/*P*_0_) = 0.993. The micropore volume was calculated using the Dubinin–Radushkevich method.^30^ The mesopore volume was calculated by subtracting the micropore volume from the total pore volume.

#### 2.2.2 X-ray diffraction measurements

X-ray diffraction (XRD) measurements were performed using a PANalytical X'Pert PRO instrument in reflection mode (Bragg–Brentano geometry) using monochromatic Cu Kα radiation (*λ* = 1.54178 Å). The operating conditions included using an anode voltage of 40 kV and an emission current of 20 mA. All measurements were recorded at room temperature. For the variable temperature powder X-ray diffraction (VT-PXRD) measurements, a XRK 900 furnace stage (Anton-Parr) was used. Samples were heated to a predetermined temperature with a ramp rate of 1 °C min^−1^ and with a 10 min equilibration time. Samples collected under vacuum were maintained using a high vacuum Oerlikon Leybold Vacuum pump PT50 kit.

#### 2.2.3 Thermogravimetric analysis

We conducted thermal stability analyses using a Netzsch TG209 F1 Libra thermogravimetrical analyser. Approximately 25 mg of sample was used in the analysis. The sample was heated from 30 °C to 900 °C at a ramp rate of 10 °C min^−1^ under N_2_ gas flow (100 mL min^−1^).

#### 2.2.4 X-ray photoelectron spectroscopy

XPS was conducted using a Thermo Scientific K-Alpha^+^ X-ray photoelectron spectrometer which is equipped with an MXR3 Al Kα monochromated X-ray source (*hν* = 1486.6 eV). The X-ray gun power was set to 72 W (6 mA and 23 kV). Prior to analysis, the samples were degassed by heating the samples at ∼0.2 mbar for 16 h at 120 °C. For sample loading, the samples were ground and mounted on the sample holder using conductive carbon tape. Before acquiring high-resolution data, a survey scan was performed using 200 eV pass energy, 0.5 eV step size and 100 ms (50 ms × 2 scans) dwell times. High-resolution N 1s, C 1s, O 1s, Cl 2p, Cu 2p and Ni 2p spectra were collected using 20 eV pass energy and 0.1 eV step size. The adventitious carbon (C–C) peak position at 285.0 eV was used as reference to shift the XPS spectra to align with this value. XPS data analysis was performed using Thermo Avantage.

#### 2.2.5 Inductively coupled plasma mass spectrometry

Inductively coupled plasma mass spectrometry (ICP-MS) analysis was conducted using an Agilent Technologies 7900 ICP-MS. Before the measurement, the sample was digested in concentrated nitric acid (HNO_3_) before being diluted to a 2% HNO_3_ (aq) solution. An Agilent SPS4 Autosampler was used for high-throughput sample analysis, with a 0.22 μm filter used to remove any remaining solid particles before analysis. Prior to analysis, the metal concentrations were calibrated at 5, 20, 200, 500 and 1000 ppb concentration level using an Aristar ICP calibration standard containing 23 transition metals. Yttrium was used as an internal standard to improve the accuracy and repeatability of the measurements. Three blanks (2% HNO_3_) were prepared for analysis to eliminate any background noise. For each material analysed, three samples were prepared, and three measurements were performed on each sample for error analysis.

### 2.3 *In situ* characterisation of Cu impregnated M-MOF-74

#### 2.3.1 X-ray absorption fine structure (XAFS) analysis

*In situ* XAFS measurements of Cu@Ni-MOF-74 and Cu@Co-MOF-74 during the Cu reduction procedure and CO adsorption were performed at B18 beamline at Diamond Light Source. From these measurements, X-ray absorption Near Edge spectroscopy (XANES) and Extended X-ray Absorption Fine Structure (EXAFS) data was extracted. Full details of the sample preparation and activation (incl. Cu reduction conditions), data acquisition and data analysis can be found in the ESI, Section 1.3.†

#### 2.3.2 Diffuse reflectance infrared Fourier Transform spectroscopy analysis

*In situ* diffuse reflectance infrared Fourier Transform spectroscopy (DRIFTS) was performed on a Bruker Tensor 27 spectrometer using a liquid N_2_ cooled mercury–cadmium–telluride (MCT) detector. Sample activation was performed by flowing He at 250 °C for 4 h with a flow rate of 12 mL min^−1^. In the case of Cu@Ni-MOF-74, to ensure Cu reduction, the sample was heated for a further 9 h at 250 °C with 10% CO in He at a flow rate of 12 mL min^−1^. After cooling to room temperature, CO adsorption was performed using 10% CO in He, and was allowed to equilibrate until saturated. Following this, excess CO was removed from the cell using He.

### 2.4 Dynamic adsorption measurements

The Cu impregnation and reduction procedure was optimised using flux response technology (FRT). This allowed us to calculate the dynamic CO adsorption capacity after *in situ* Cu reduction and evaluate the Cu@M-MOF-74 CO purification performance. We conducted binary CO/N_2_ and CO/CO_2_ adsorption measurements using a breakthrough apparatus to test the best performing Cu@Ni-MOF-74 sample.

#### 2.4.1 Flux response technology

The fundamentals and theory of this technique are explained by Palmer *et al.*,^31^ with the apparatus schematic shown in Fig. S1.† In brief, the experimental set-up replicates that of a Wheatstone bridge assembly, where a system side and reference side are used as different legs of the bridge. The perturbation gas entering the system side causes an imbalance of the bridge assembly due to pressure fluctuations. The differential pressure between the system and reference side were measured using a differential pressure transducer (manufactured by Furness Controls, model R.S.N 9411283), with data acquisition using a Data/Shuttle USB 54 system. The flow-rates were controlled by Bronkhorst mass-flow controllers. Delay lines (1/4′′ OD PFA tubing) were installed to improve the data resolution upon gas switching.

Samples (∼80 mg of dry sample) were loaded in a 4 mm ID quartz tube and packed using quartz wool. The column was pressurised and sample activation and Cu reduction was performed depending on the requirements, outlined below. After activation and reduction, the He and CO line was switched to N_2_ to purge and remove He and CO in preparation for analysis. For analysis, the CO flow-rate was set to 5 mL min^−1^ (N_2_ flow-rate at 10 mL min^−1^) and the CO perturbation gas was switched using the 3-port valve. An adsorption peak was recorded along the new baseline due to the pressure response upon adsorption. After equilibration, the system side flow-rate was measured using a bubble flow-meter, and the CO perturbation gas was then switched off to induce desorption.

An example of an FRT profile is shown in Fig. S2.† The integrated area under adsorption peak (with aid of the curve fitting tool on Origin) was subtracted from an area of the graph with a known quantity of flowing adsorbate molecules after perturbation. This provided the number of adsorbed molecules from which the dynamic CO adsorption capacity could be determined. The standard deviation for measurements on the instrument is ±5%, and error also arises from experimental variance of the MOF synthesis and Cu impregnation and reduction procedure.

##### 2.4.1.1 Cu impregnation loading and Cu salt precursors analyses

For M-MOF-74 and Cu@M-MOF-74 (M = Ni or Co) activation and Cu^2+^ to Cu^+^ reduction, the samples were heated for 6 h at 250 °C with 10% CO (in He) flowing at 1 bar on the FRT instrument.

##### 2.4.1.2 Cu reduction analysis

For Ni-MOF-74 activation and Cu^2+^ to Cu^+^ reduction, Ni-MOF-74 and Cu@Ni-MOF-74 samples were subjected to three different activation and Cu reduction procedures: (i) heating at 250 °C with He flowing for 16 h, (ii) heating at 250 °C with He flowing for 16 h, followed by 10% CO (in He) at 250 °C for 6 h and (iii) heating at 250 °C with 10% CO (in He) for *x* h (where *x* = 2, 6 and 16).

#### 2.4.2 Binary gas breakthrough column testing

The apparatus schematic of the home-built breakthrough column system is shown in Fig. S3,† and the experimental procedure is outlined below. The full formula derivation for the breakthrough adsorption capacity and selectivity calculations can be found in the ESI, Section 1.5.2.†

Samples (∼200 mg) were loaded in a 4 mm ID quartz tube and packed using quartz wool. The packed tube was loaded and the system pressurized to 1 bar gauge pressure. Sample activation and Cu reduction was performed by flowing 10% CO in He with a total flow-rate of 20 mL min^−1^ for 6 h at 250 °C using heating tape (Omega). The temperature was controlled using a temperature probe (RS components) and a Proportional–Integral–Derivative (PID) controller.

For analysis, the volumetric flow-rates (total flow-rate of 20 mL min^−1^) were set for 50 : 50 v/v CO : X (X = N_2_, CO_2_) mixture composition and a back-pressure regulator was used to control the inlet feed pressure. The flow-rates were controlled by Bronkhorst mass-flow controllers and outlet flow-rate controlled by a Bronkhorst mass-flow meter. The four-port switch valve was switched to send the binary mixture to the sample column before reaching the MS detector at the outlet.

Analysis of the outlet feed concentration was performed by an ESS GeneSys Quadrupole Mass Spectrometer (MS) 200, with data acquisition using a Quadstar 32 bit software. Digital pressure gauges at the inlet and outlet were used to monitor the pressure drop in the column, which did not exceed 0.1 bar. Hexagonal boron nitride (Saint Gobain, Tres BN) was used as a non-adsorbing material to measure the dead volume of the instrument using He. The breakthrough curves have been normalised to eliminate the inlet dead volume time, so the plotted curves are a direct reflection of dynamic adsorption of the given sample. The relative concentration, *C*/*C*_0_, on the *y*-axis is standardised to the mole fractions of the inlet feed. Here, the breakthrough capacity (adsorption uptake when *C*/*C*_0_ = 0.01) is presented as opposed to the saturation capacity (adsorption uptake when *C* = *C*_0_). An industrial adsorption cycle would not enable the adsorbent bed to reach equilibrium with the inlet feed, therefore breakthrough capacity is a more useful metric. The standard deviation for measurements on the instrument is ±5%, and error also arises from experimental variance of the MOF synthesis and Cu impregnation and reduction procedure.

## 3. Results and discussion

We impregnated Ni-MOF-74 and Co-MOF-74 with Cu and carried out a systematic Cu impregnation study, with the aim to produce an improved CO purification adsorbent. We present our findings below.

### 3.1 Characterisation of Cu impregnated samples

Initially, we synthesized Cu@Ni-MOF-74 samples using CuCl_2_ and Cu(HCO_2_)_2_ as Cu precursors and used the results to optimise the Cu loading. We selected CuCl_2_, a commonly used precursor,^17,18,21–23^ because of its ability to be reduced to CuCl leading to strong Cu^+^–CO π-complexation. Cu(HCO_2_)_2_ was chosen due to its ability to simultaneously provide a reducing agent and a Cu^+^ source.^21,22^ Indeed, upon heating, the decomposition of the formate ion produces H_2_ for Cu^2+^ to Cu^+^ reduction.^32^

We initially impregnated Ni-MOF-74 with equimolar CuCl_2_ : Cu(HCO_2_)_2_ ratios while varying the Cu loading. We used ICP-MS and XPS analyses to quantify the loading (Table S2 in the ESI, Section 3.1†). ICP-MS probed the bulk Cu loading, while XPS measured the surface loading and as a result, the values from XPS analyses surpass those from ICP-MS. From the ICP-MS analysis, the estimated Cu loadings are 1.77, 3.50 and 7.07% wt and the samples are subsequently referred to as 2-, 4- and 7-Cu@Ni-MOF-74 throughout. These percentages represent the ratios of Cu over Ni in the samples.

**Fig. 1:**
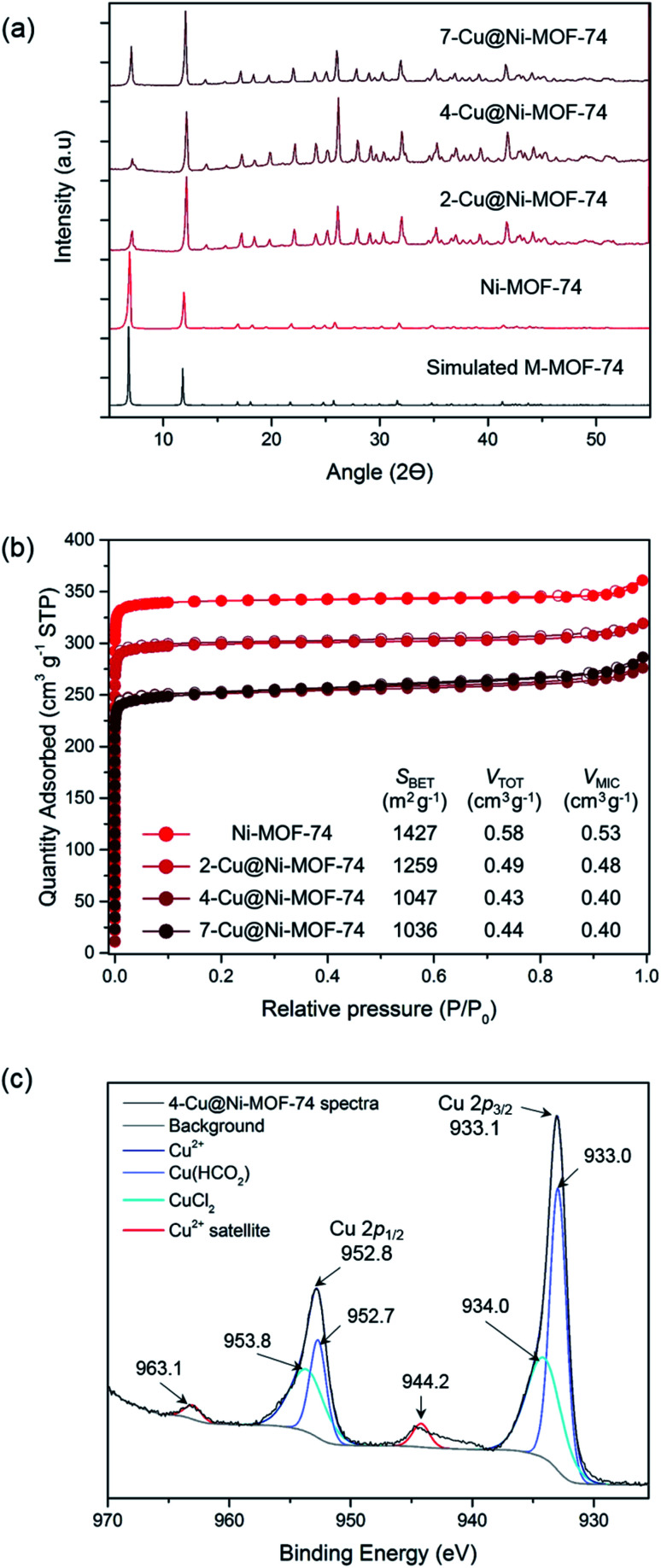
Characterisation of the Cu-impregnated MOFs: (a) X-ray diffraction patterns of simulated MOF-74, Ni-MOF-74, 2-Cu@Ni-MOF-74, 4-Cu@Ni-MOF-74 and 7-Cu@Ni-MOF-74. (b) N_2_ sorption isotherms at −196 °C of Ni-MOF-74, 2-Cu@Ni-MOF-74, 4-Cu@Ni-MOF-74 and 7-Cu@Ni-MOF-74. The textural parameters derived from the isotherms are shown on the table. (c) Cu 2p spectra of 4-Cu@Ni-MOF-74. The deconvoluted Cu peaks and energy levels are shown alongside the characteristic Cu^2+^ satellites.

Using XRD, we confirmed that the impregnation did not significantly alter the MOF structure and that there was no major Cu salt particle aggregation (Fig. 1(a)). Porosity measurements allowed us to study the effect of impregnation on pore blocking with increasing Cu amount (Fig. 1(b) and Table S2†). Increasing Cu loading reduced the BET surface area and pore volume as anticipated, due to the Cu sites inducing pore blocking and reducing accessibility to pores. As the Cu loading approximately doubled from 4- to 7-Cu@Ni-MOF-74, there was no additional reduction in porosity. This observation suggests that Cu accumulated on the external surface beyond 3.50% wt Cu loading.

VT-PXRD and TG analyses enabled us to establish an activation and reduction temperature that would not damage the Ni-MOF-74 and Co-MOF-74 structures (see ESI, Fig. S4–S6†). The thermogravimetric curves indicated solvent release <350 °C, thermal decomposition at 350–400 °C and total decomposition at ∼550 °C. The VT-PXRD spectra (Fig. S5 and S6†) pointed to MOF degradation at 400–450 °C. Therefore, we chose 250 °C for this study to maintain structure stability during the thermally-activated Cu reduction.

We confirmed the oxidation state of surface Cu post activation and reduction using XPS. The full XPS survey of 4-Cu@Ni-MOF-74 is shown in Fig. S7.†Fig. 1(c) shows the XPS 2p spectra of 4-Cu@Ni-MOF-74. The reference 2p spectra of the Cu salts are presented in Fig. S8.† The Cu 2p subshell is split into doublets: 2p_3/2_ and 2p_1/2_. The presence of Cu(CHO_2_)_2_ and CuCl_2_ is confirmed by comparison against the spectra of the pure Cu salts. These peaks are assigned on the basis that the maximum Cu 2p_3/2_ and Cu 2p_1/2_ peaks of the CuCl_2_ Cu 2p XPS spectra (Fig. S8†) are more energetic than the Cu(CHO_2_)_2_ Cu 2p peaks. The negative charge of the oxygen formate ions is dispersed across two atoms and therefore the CuCl_2_ chloride ions result in a slightly more energetic Cu 2p peaks. The small peaks at 944.2 and 963.1 eV in Fig. 1(c) are due to the characteristic Cu^2+^ satellite peaks.^16^ Their weak intensities result from Cu^2+^ reduction to Cu^+^ (or possibly Cu^0^) facilitated by the heat treatment under vacuum during sample preparation. Overall, the XPS results indicate the possible reduction of Cu^2+^ to Cu^+^ but they are only representative of the surface chemistry of the materials. The bulk chemistry of the materials is investigated later using XANES.

### 3.2 Effect of Cu impregnation on CO adsorption

Having produced Cu@Ni-MOF-74 and Cu@Co-MOF-74 samples of varying Cu loadings, we then evaluated their dynamic CO capacity using FRT. This technique allowed us to activate and reduce the samples *in situ* under flowing He or CO atmosphere prior to testing.

#### 3.2.1 Cu loading analysis

**Fig. 2:**
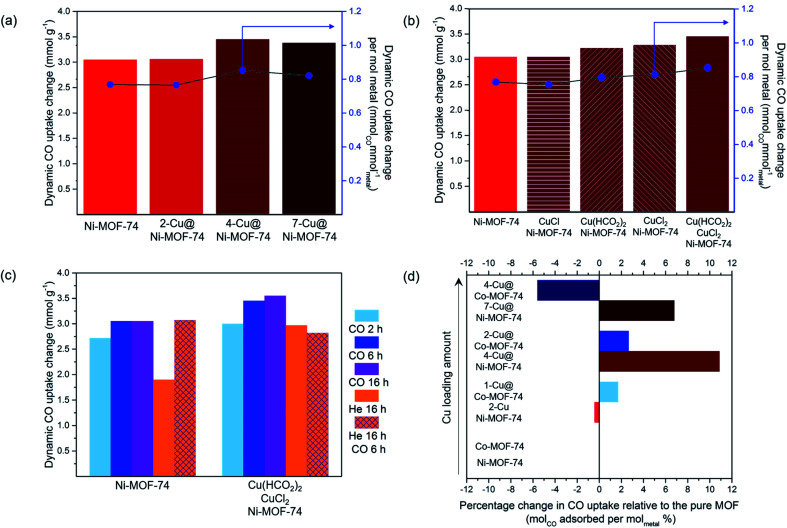
Dynamic CO capacities (50 : 50 v/v CO : N_2_) measured at 25 °C and 1 bar on the FRT set-up: (a) effect of Cu loading; (b) effect of the type of Cu salts; (c) effect of activation and reduction procedures; (d) case of Cu@Co-MOF-74.

We conducted this analysis on the Cu@Ni-MOF-74 samples. The Ni-MOF-74 activation and Cu reduction conditions are outlined in Section 2.4.1.1, and their dynamic CO capacities (50 : 50 v/v CO : N_2_) are presented in Fig. 2(a), 25 °C and 1 bar. The uptakes are presented per gram of Ni-MOF-74 and Cu@Ni-MOF-74. Overall, Cu impregnation brings limited gains and appears to be detrimental over 3.50% wt Cu loading, as the difference in values from unimpregnated Ni-MOF-74 falls within the ±5% standard deviation. The increased quantity of Cu induces pore blockage and reduces its capacity. Furthermore, some Cu^2+^ may remain and limit access to the more strongly binding Ni^2+^ open-metal sites.

To accurately study the influence of the Cu loading, one must consider the increased metal density of the structure. Therefore, we normalised the CO uptake per mol of metal in the adsorbent, taking into account both the Cu loading and the Ni content (Table S2†). The results normalised to Cu only are also presented in Table S2.†

Thermal activation and Cu reduction were performed for 6 h at 250 °C with flowing 10% CO (in He) unless stated otherwise. The dynamic CO capacity per mol of metal was calculated using the measured values on the FRT and the Cu loading obtained from the ICP-MS analysis. (a) Dynamic CO capacities at varied Cu loadings using equimolar CuCl_2_ : Cu(HCO_2_)_2_ salt precursors. (b) Dynamic CO capacities using different Cu salts for impregnation. (c) Dynamic CO capacities using CuCl_2_ : Cu(HCO_2_)_2_ salts with thermal activation and Cu reduction performed using He or CO atmosphere. (d) Dynamic CO capacities of Cu@Ni-MOF-74 and Cu@Co-MOF-74 at varied Cu loadings using equimolar CuCl_2_ : Cu(HCO_2_)_2_ salt precursors.

These results demonstrate that increasing the Cu loading above 3.50% wt is detrimental (Fig. 2(a) and Table S2†). The normalised values show 4-Cu@Ni-MOF-74 (3.50% wt) achieves the highest CO uptake per gram of metal and per mol of Cu. Increasing Cu loading above 3.50% wt reduces the normalised CO uptake. For this reason, we identified 4-Cu@Ni-MOF-74 as the optimised Cu loading for Ni-MOF-74.

#### 3.2.2 Cu salts analysis

After determining the optimum range of Cu loading, we studied the effect of using different Cu salts for Cu impregnation (*i.e.* CuCl_2_, Cu(HCO_2_)_2_ and CuCl or a combination thereof) of Ni-MOF-74. The Ni-MOF-74 activation and Cu reduction conditions are outlined in Section 2.4.1.2. We normalised the dynamic capacities to the Cu loading to account for the small differences in Cu loading (3.17 to 3.95% wt). The results, presented in Fig. 2(b), show the presence of CuCl_2_/Cu(HCO_2_)_2_ assists the reduction procedure, leading to the highest CO capacity, though only slightly. Impregnation with solely Cu(HCO_2_)_2_ produces the second highest CO capacity, indicating CuCl_2_ is required as another source of Cu to improve CO capacity. The decomposed residual carbon of the formate precursor adds to the specific weight of the adsorbent. This fraction of the adsorbent mass did not contribute to CO adsorption. CuCl impregnation exhibited similar CO uptake to ‘pure’ Ni-MOF-74, and a small reduction in the normalised CO uptake. This result is due to Cu^+^ to Cu^0^ reduction upon heating with CO. To summarise, impregnation with a mixture of CuCl_2_ and Cu(HCO_2_)_2_ leads to the highest CO uptake.

#### 3.2.3 Cu reduction analysis

The activation and Cu reduction procedure through heat treatment was investigated using He and CO atmosphere for increasing durations (conditions outlined in section 2.4.1.3). We conducted this analysis on the 4-Cu@Ni-MOF-74 samples. The CO dynamic uptakes for the various activation and reduction procedures are shown in Fig. 2(c).

Activating Ni-MOF-74 under CO atmosphere produced a higher dynamic CO capacity than activating under He. We hypothesize that at elevated temperatures, CO reacts with residual MeOH bound to the MOF, thereby increasing the number of open-metal sites.^33^ Activation *via* a combination of heating and CO flow led to the highest CO uptake for the equimolar CuCl_2_ : Cu(HCO_2_)_2_ impregnated sample. Overall, the results indicate that both He and CO behave as reducing agents to different degrees, and dictate the duration of heat treatment required. Following this study, we chose to conduct Cu^2+^ to Cu^+^ reduction *via* heating at 250 °C with 10% CO (in He) for 6 h as the activation and Cu reduction procedure for the subsequent experiments. These conditions compromise between minimising the time of CO used at elevated temperature while maximising the amount of Cu^+^ generated.

#### 3.2.4 Cu@Co-MOF-74 analysis

We now turn our attention to the Cu@Co-MOF-74 samples and the impact of Cu impregnation on CO uptake (Fig. 2(d)). We employed the equimolar CuCl_2_ : Cu(HCO_2_)_2_ mixture for impregnation. The ICP-MS analyses indicated Cu loading of 1.28, 2.42 and 4.22% wt The corresponding samples are referred to as 1-, 2- and 4-Cu@Co-MOF-74. The CO uptakes were normalised to account for the variance in Cu loading. 1-Cu@Co-MOF-74 and 2-Cu@Co-MOF-74 adsorbed similar amounts of CO, with marginal improvement compared to Co-MOF-74 (Fig. 2(d)). Cu loading above 2.42% wt was detrimental due to pore blocking and reduced accessibility to framework open-metal sites for strong Co^2+^–CO binding. We attribute the differences in uptake limit between Co- and Ni-MOF-74 to differences in particle size and defect sites, as shown by the unexpected measured mesopore volume of Ni-MOF-74 (Table S2†). 4-Cu@Ni-MOF-74 and 2-Cu@Co-MOF-74 exhibit similar degrees of reduction in porosity (Table S2†) compared to the unimpregnated samples, excluding any porosity effect upon these discrepancies.

### 3.3 *In situ* Cu@M-MOF-74 XAFS characterisation

XAFS experiments were conducted on the best performing Cu impregnated M-MOF-74 samples, *i.e.* 4-Cu@Ni-MOF-74 and 2-Cu@Co-MOF-74. The objectives were: (i) to investigate further the Cu reduction step since XPS only provided information on surface Cu sites, (ii) to assess the impact of the Cu impregnation on the states of the Ni and Co sites and (iii) to monitor changes in the Cu, Ni and Co sites upon CO adsorption.

#### 3.3.1 XAFS analysis of the Cu species during the activation and reduction procedure

**Fig. 3:**
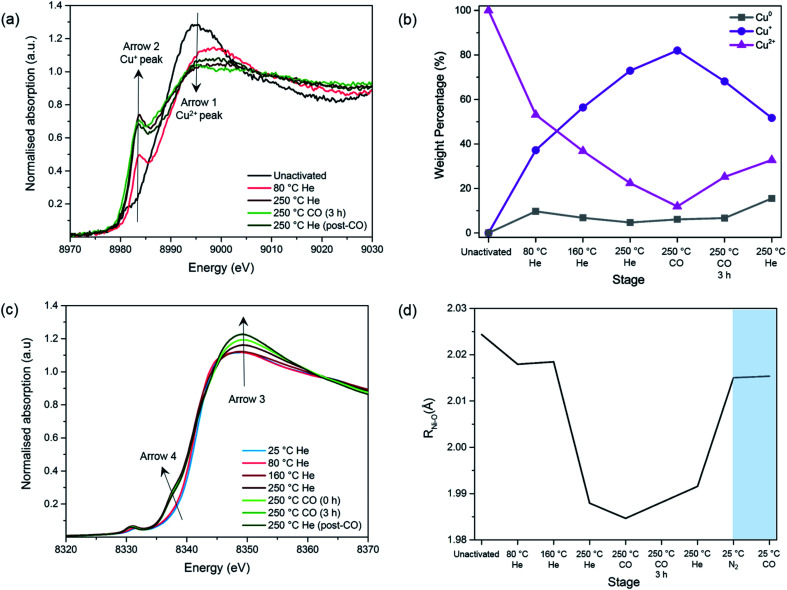
Study of the MOF activation and Cu reduction procedure of 4-Cu@Ni-MOF-74: (a) Cu-edge XANES spectra. Arrow 1 = Cu^2+^ peak; Arrow 2 = Cu^+^ peak. (b) Linear Combination Fit analysis of Cu species. (c) Ni-edge XANES spectra. (d) Ni–O bond lengths obtained from the EXAFS fit during the activation and Cu reduction procedure and the room temperature adsorption of N_2_ and CO (shaded). The average error for the bond length is 0.01 Å.

Here, the Cu reduction process was evaluated upon a progressive activation and reduction procedure. The activation and reduction procedure consisted in a progressive heating (10 °C min^−1^) under He up to 250 °C, followed by a switch to a CO atmosphere and switching back to He accompanied with cooling to 25 °C. The Cu-edge XANES spectra for 4-Cu@Ni-MOF-74 are shown in Fig. 3(a), S10 and S11.† Those for 2-Cu@Co-MOF-74 are shown in Fig. S12.†

Before the activation/reduction process, 4-Cu@Ni-MOF-74 exhibits the characteristic intense white line peak of Cu^2+^ (*i.e.* intense absorption in the near-edge) at 8995 eV (Fig. 3(a)). As the temperature increases, the intensity of the white line decreases and is accompanied by the appearance of a feature at 8983.7 eV. This feature is attributed to the first transition peak of Cu^+^, and is confirmed when its first derivative is overlaid with the CuCl standard (Fig. S13†). This observation indicates reduction from Cu^2+^ to Cu^+^ through oxidation of the formate (eqn (1) and (2)) proceeds at temperatures as low as 80 °C.

1 2COOH^−^ ↔ H_2_ + CO_2_

2 2Cu^2+^ + H_2_ ↔ 2Cu^+^ + 2H^+^

By applying a linear combination fit in Athena, similar to that used by Lamberti *et al.*,^34^ we determined the percentage of each Cu species present (Cu^2+^, Cu^+^ and Cu^0^) at various stages of activation and reduction using the spectra from the pure standards as references (Fig. 3(b)). Overall, as the temperature increases, the amount of Cu^+^ increases while that of Cu^2+^ decreases. At the end of the activation/reduction procedure, some Cu^2+^ species are present (32.8%) but the majority of the copper is Cu^+^ (51.7%), with a small but significant amount of Cu^0^ (15.5%) due to over reduction. We hypothesize that disproportionation of Cu^+^ also occurs, as the amount of Cu^2+^ increases when flowing with pure CO and He at 250 °C.

A switch from a pure flow of He to a pure flow of CO at 250 °C decreases the intensity of the peak at 8983 eV (see Fig. S11†). This observation is attributed to the formation of [Cu^(I)^(CO)_*n*_]^+^ adducts in the sample, as previously shown by Prestipino *et al.* and Yamashita *et al.* in the case of Cu^+^ in ZSM-5.^35,36^ The formation of these adducts reduces the amount of quantifiable Cu^+^ species, explaining the decrease of the Cu^+^ peak upon the introduction of CO at 250 °C (Fig. 3(b)). However, as reduction proceeds the CO adduct decomposes and the Cu^+^ peak at 8983 eV increases again (Fig. S11†).

A similar analysis of the Cu^+^ evolution through the activation/reduction process was performed for the Co-MOF-74 based samples and similar conclusions are derived. The amount of Cu^0^ species present for this sample remains negligible (Fig. S14†).

#### 3.3.2 XAFS analysis of the Ni and Co species during the activation and reduction procedure

Following a greater understanding of the Cu reduction process, the interactions of the impregnate with the MOF metal centres were investigated. This study is important as Co^2+^ and Ni^2+^ centres of MOF-74 strongly bind to CO. Any blockage or interference of these sites could be detrimental to the adsorption process. The Ni-edge XANES spectra of 4-Cu@Ni-MOF-74 and Co-edge XANES spectra of 2-Cu@Co-MOF-74 during the reduction process can be seen in Fig. 3(c) and S15,† respectively.

The following changes are observed as the temperature and the gas composition change: (i) an increase in the intensity of the white line position at ∼8348 eV with a shift towards higher energies (Arrow 3), (ii) the appearance of a pre-edge feature assigned to 1s–4p_*z*_ transition at 8338 eV (Arrow 4) and (iii) an increase in intensity of the pre-edge feature with a shift towards lower energies assigned to 1s–3d transition at 8333 eV. These observations are mostly coherent with the previously reported results regarding the pre-edge feature of the Ni- and Co-edge of the MOFs during heating.^37,38^ During MOF activation, the symmetry changes from octahedral to square-pyramidal as the solvent and water molecules attached to the Ni^2+^ and Co^2+^ open-metal sites desorb and provide accessibility for CO adsorption. This causes the removal of the degeneration of p levels and the loss of the inversion centre^37,38^ and allows for the transition 1s–4p_*z*_ to appear,^39,40^ as well as for the increase in intensity in the 1s–3d electronic transition which is forbidden in a perfect octahedral symmetry.^39,41^

#### 3.3.3 EXAFS analysis of the Ni and Co species geometry during the activation and reduction procedure and N_2_ and CO adsorption at room temperature

We used EXAFS analysis to examine the geometry and bond lengths of the MOF structures during activation and Cu reduction and the room temperature adsorption of N_2_ and CO. The results are presented in Fig. 3(d) for 4-Cu@Ni-MOF-74 and Fig. S16† for 2-Cu@Co-MOF-74. A decrease in the M–O distance is observed upon heating, with further decrease upon introduction of CO at 250 °C. These contractions indicate a change in the MOF structure from an octahedral to square pyramidal geometry as non-linker molecules coordinated to the metals are released. The observations align with previous studies regarding the configuration of M-MOF-74.^38,42^

Upon addition of N_2_, the M–O bond length increases from 1.991 to 2.015 Å for Ni–O and from 2.014 to 2.044 Å in the case of Co–O (Fig. S16†). This suggests that the system has moved back to a 6-fold coordination state.^38,43^

Upon addition of CO, no further changes can be seen, which is in disagreement with the previously reported data.^43^ This could be due to the short *k*-range of the data that does not allow a fine distinction between the O atoms of the ligands and the CO species. To summarise, we observed no unexpected trends of the framework geometry, demonstrating negligible interference from the Cu species in the adsorption of CO on the Ni and Co sites.

#### 3.3.4 XAFS analysis of the Cu species during N_2_ and CO adsorption

**Fig. 4:**
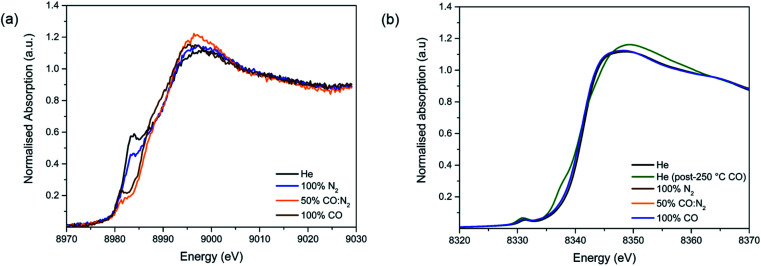
Study of CO adsorption on 4-Cu@Ni-MOF-74: (a) Cu-edge XANES spectra. (b) Ni-edge XANES spectra.

We now focus on monitoring the state of the Cu sites upon exposure to CO and N_2_ for adsorption. The results for the Cu-edge of 4-Cu@Ni-MOF-74 are presented in Fig. 4(a) and S17† and those for 2-Cu@Co-MOF-74 in Fig. S18.† As N_2_ is introduced, the white line at 8982 eV decreases (Fig. S17†), suggesting an interaction between N_2_ and Cu^+^. Drake *et al.* reported this behaviour for Cu^+^ interacting with other molecules.^44^

Upon the addition of CO, the white line intensity decreases and shifts towards lower energies (Fig. S18†). This pattern has been previously seen for CO and NO interacting with Cu^+^ cations in zeolites. The results suggest much stronger interactions between Cu^+^ and CO than the one with N_2_ as in a 50 : 50 N_2_ : CO mixture the only ligand present appears to be CO.

#### 3.3.5 XAFS analysis of the Ni and Co species during N_2_ and CO adsorption

Parallel to the study of the Cu sites, the states of the Ni and Co sites were also monitored upon N_2_ and CO adsorption. The results for 4-Cu@Ni-MOF-74 are presented in Fig. 4(b) and those for 2-Cu@Co-MOF-74 in Fig. S19.† The interaction of the MOF with N_2_ immediately restores the *O*_h_-like symmetry around the Ni^2+^ and Co^2+^ centres, as observed by the disappearance of the feature at 8338 eV and the decrease of the white line at 8348 eV to the intensity of the solvated material. This confirms Ni^2+^–CO and Co^2+^–CO binding and demonstrates the negligible effect of Cu impregnation upon the Ni^2+^–CO and Co^2+^–CO interactions.

### 3.4 *In situ* Cu@M-MOF-74 DRIFTS characterization

**Fig. 5:**
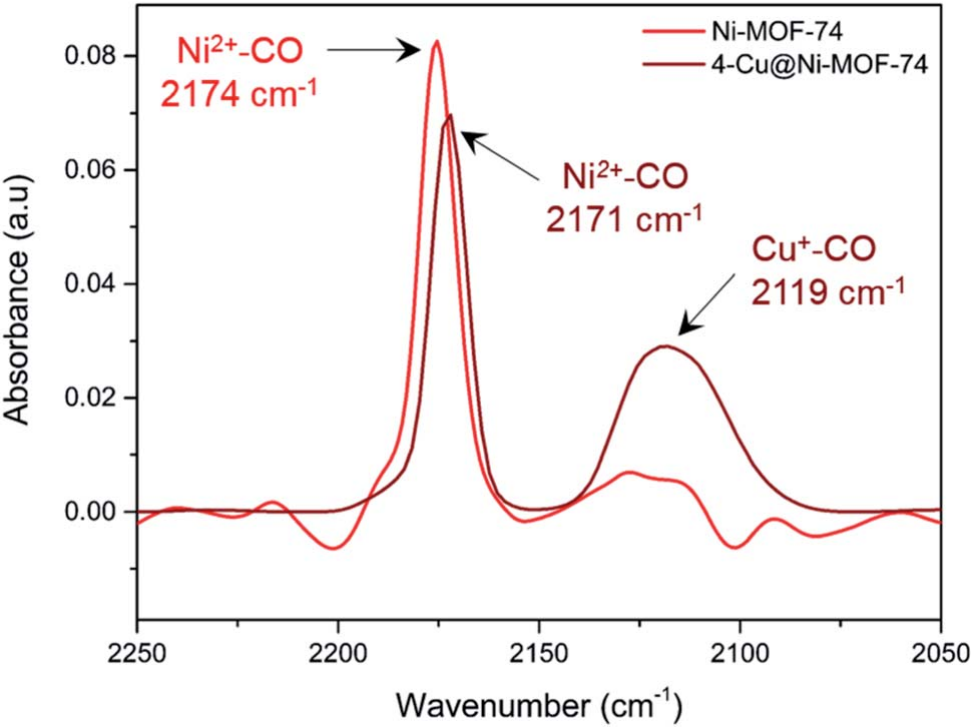
*In situ* DRIFTS measurement of chemisorbed CO on Ni-MOF-74 and 4-Cu@Ni-MOF-74 at 25 °C and 1 bar. Each spectrum has been background corrected with respect to the instrument and sample holder.

We used diffuse reflectance infrared Fourier Transform spectroscopy (DRIFTS) to further study the metal–CO interactions of both Ni and Cu of 4-Cu@Ni-MOF-74 and pure Ni-MOF-74 (Fig. 5). We consider first the 4-Cu@Ni-MOF-74 sample. The Ni^2+^–CO stretch appears at 2171 cm^−1^, and corresponds to a non-classical interaction.^7^ The spectrum shows that Cu^+^ impregnation left the framework Ni^2+^ sites unaffected and accessible to CO binding. This aligns with the findings from the XAFS study. We observe Cu–CO interaction at 2119 cm^−1^. A free CO stretch would be expected 2143 cm^−1^, therefore the band at 2119 cm^−1^ indicates a classical Cu^*x*+^–CO interaction.^45^ This stretch is assigned to [Cu^(I)^(CO)]^+^ species, previously observed in Cu-ZSM-5 and at defect sites in Cu-MOF-74.^46,47^ Ni-MOF-74 spectrum is similar to that of 4-Cu@Ni-MOF-74, minus the Cu–CO interactions. We also note that the small band observed at 2115–2130 cm^−1^ is part of the background noise.

### 3.5 Dynamic CO/N_2_ and CO/CO_2_ separation testing

**Fig. 6:**
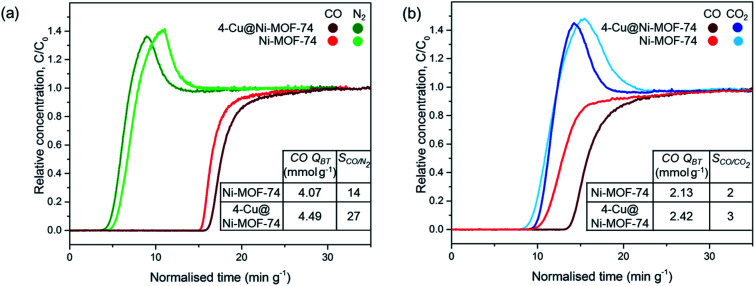
Breakthrough curves of Ni-MOF-74 and 4-Cu@Ni-MOF-74 for 50:50 v/v binary gas mixtures on a breakthrough adsorption column at 25 °C and 1 bar: (a) CO : N_2_ mixture and (b) CO : CO_2_ mixture. (CO *Q*_BT_ = CO breakthrough capacity, *S*_CO/X_ = CO/X ideal selectivity at equilibrium where X = N_2_ or CO_2_).

We tested the dynamic separation performance of 4-Cu@Ni-MOF-74 for CO/N_2_ and CO/CO_2_ using a dynamic breakthrough column apparatus. We compared the results to those of Ni-MOF-74 to evaluate the effect of Cu impregnation. The breakthrough curves for 50 : 50 v/v binary mixtures are shown on Fig. 6.

For the 50 : 50 v/v CO : N_2_ mixture, N_2_ breaks through first at similar times for both samples because of the weaker physisorption forces responsible for adsorbing to non-polar N_2_. Upon CO breakthrough, the N_2_ concentration exceeds the inlet feed concentration and produces a roll-up effect. The competitive binding with CO induces a spike in concentration as CO replaces the weakly adsorbed N_2_ molecules. Cu impregnation resulted in an increased breakthrough capacity (from 4.07 to 4.49 mmol g^−1^) and CO/N_2_ selectivity at equilibrium after saturation (from 14 to 27). The increase in CO/N_2_ separation performance is attributed to: (i) the increased number of open-metal sites binding to CO and (ii) the lower porosity of the Cu impregnated samples reducing N_2_ sorption.^21^ Introducing Cu^+^ sites with no affinity to N_2_ results in greater CO/N_2_ selectivity. We did not observe the same degree of improvement earlier with the FRT. Indeed, in the FRT tests, the sample is pre-saturated with N_2_ before CO adsorption, and the instrument is unable to distinguish the CO selectivity and separation performance.

Fig. 6(b) exhibits similar breakthrough times for CO and CO_2_ for Ni-MOF-74 because of similar binding strength between the open-metal sites and polar CO and CO_2_ adsorbates. The CO breakthrough capacity is smaller than the amount in a CO/N_2_ stream because of the MOFs greater affinity to CO_2_ over N_2_.^21,22^ Cu impregnation improved the CO/CO_2_ separation performance by increasing the number of Cu^+^ open-metal sites which undergo strong π-complexation to CO, but do not interact with CO_2_ through this mechanism.^22^ Therefore, both the CO breakthrough capacity and CO/CO_2_ selectivity increased upon impregnation. Cu impregnation replaced the accessibility of Ni^2+^ open-metal sites for CO and CO_2_ binding with Cu^+^ open-metal sites solely for CO binding. CO/N_2_ and CO/CO_2_ separation performance was also improved after Cu impregnation of Fe-MIL-100 and Cr-MIL-101,^21–26^ but was improved to a smaller degree with M-MOF-74 because of the highly active Ni^2+^ sites compared to Fe^3+^ and Cr^3+^.

In an industrial setting, an adsorbent must maintain high adsorption capacity and selectivity over multiple cycles and require minimal energy for regeneration. Previous studies have tested the cyclability of Ni-MOF-74 (ref. 6) and Cu impregnated MOFs for CO purification,^22,23,25^ with He purging^22^ and vacuum swing^25^ regeneration techniques favoured to minimise any potential overreduction which would be associated with thermal regeneration. Yet, vacuum swing and He purging would not regenerate the Ni^2+^ sites of the MOF. A high regeneration temperature is required due to the high CO isosteric heat of adsorption value of Ni-MOF-74 (ref. 6) (−54.4 kJ mol^−1^ at low loading) potentially leading to undesired chemisorbing Cu^0^ sites. Hence, further monitoring and optimisation of the Cu oxidation state is needed for cyclic testing with varying regeneration conditions, while simultaneously measuring its CO separation performance.

## 4. Conclusion

We have optimised the Cu impregnation procedure for M-MOF-74 (M = Ni or Co) to improve the CO purification ability of the adsorbent. The optimisation has focused on identifying the type of Cu salt, the Cu loading as well as the MOF activation and Cu reduction process required to maximise CO uptake. A mixture of CuCl_2_ and Cu(HCO_2_)_2_ salts with a 4% wt Cu loading led to the largest CO uptake. In terms of activation and Cu reduction, we have established that heating at 250 °C with 10% CO (in He) for 6 h allowed the best compromise between minimising the time of CO used at elevated temperature while maximising the amount of Cu^+^ generated.

We have performed *in situ* XAFS analysis of the Cu impregnated Ni- and Co-MOF-74 samples during the activation/reduction procedure as well as during CO adsorption. This type of *in situ* analysis has not been performed on Cu impregnated MOFs before. Using this technique, we have shown that Cu^+^ is generated with He flowing at temperatures as low as 80 °C and becomes the dominant Cu species at the end of the activation/reduction procedure (between 57% and 70% depending on the MOF). Some Cu^2+^ and Cu^0^ still remain in the activated samples. Using this same technique, we have confirmed Cu^+^–CO binding for the first time on Cu impregnated MOFs. EXAFS analysis has confirmed no interference from the Cu species on CO adsorption on the Ni and Co sites. DRIFTS analysis has further supported this finding.

Finally, we have performed dynamic CO/N_2_ and CO/CO_2_ separation measurements 50 : 50 v/v gas mixtures (*i.e.* CO/N_2_ and CO/CO_2_) to assess any enhancement in CO purification upon Cu impregnation. CO uptake and CO/N_2_ selectivity increased by 10% and 93%, respectively, for CO/N_2_ separation. This is due to a combined increase in the number of open-metal sites for metal–CO binding and decrease in porosity reducing N_2_ sorption. CO uptake and CO/CO_2_ selectivity increased by 14% and 50%, respectively. This is due to the Cu sites preferentially interacting with CO rather than CO_2_. Overall, this study provides the first confirmation of Cu^+^–CO binding in Cu impregnated MOFs after optimising the synthesis and reduction. This impregnation was demonstrated to improve CO separation ability in CO/N_2_ and CO/CO_2_ mixtures.

## Conflicts of interest

There are no conflicts to declare.

## Supplementary Material

RA-010-C9RA10115B-s001
