# Supplementary material for: Optimisation of Cu+ impregnation of MOF-74 to improve CO/N2 and CO/CO2 separations
Source: RSC Adv. 2020 Jan 31;10(9):5152–62. doi: 10.1039/c9ra10115b (PMC9049075; doi:10.1039/c9ra10115b)
Supplement: RA-010-C9RA10115B-s001 [file RA-010-C9RA10115B-s001.pdf]

Electronic Supplementary Material (ESI) for RSC Advances.
This journal is © The Royal Society of Chemistry 2020

Supporting Information

Optimisation of Cu^+ impregnation of MOF-74 to improve CO/N_2 and CO/CO_2 separations

Arwyn Evans,[1] *Matthew Cummings,*[2] *Donato Decarolis,* [1⊥] *Diego Gianolio,*[3] *Salman Shahid,*[1⊥] *Gareth Law,*[2] *Martin Attfield,*[2] *David Law,*[4] *and Camille Petit*[1]

[1] Barrer Centre, Department of Chemical Engineering, Imperial College London, UK

[2] School of Chemistry, The University of Manchester, UK

[3] Diamond Light Source, Harwell Campus, Didcot, UK

[4] BP Chemicals Ltd Petrochemicals Technology, Saltend, Hull, UK

⊥Current affiliations:

D. Decarolis: School of Chemistry, Cardiff University, UK

S. Shahid: Centre for Advanced Separations Engineering, Department of Chemical Engineering, University of Bath, UK

1. Literature Review

1.1. CO purification performance comparison

Table S1. Summary table of MOFs and their Cu impregnated analogues reported to date for CO adsorption and CO/N_2 and CO/CO_2 separation at varying temperature and 1 bar.

**Materials**	**Temperature** (°C)	**CO uptake** (mmol g$^-$)	**N_2 uptake** (mmol g$^-$)	**CO_2 uptake** (mmol g$^-$)	**$CO/N_2$$^{\pm}$ selectivity**	**$CO/CO_2$$^{\pm}$ selectivity**	**S_{BET}** (m^2 g^{-1})	**Reference**
Cu^+-impregnated Fe-MIL-100	25	3.75	0.10		250		1067	1
Fe-MIL-100	25	0.50	0.22		10		2023	1
Cu^+-impregnated Fe-MIL-100	30	3.30	0.01	0.30	900	11*	898	2
Fe-MIL-100	30	1.05		3.60		0.3*	2458	2
Cu^+-impregnated Fe-MIL-100	25	3.10		0.20		420	790	3
Fe-MIL-100	25	0.20		2.03		0.1*	2200	3
Cu^+-impregnated Fe-MIL-100	25	2.78	0.15		180		762	4
Fe-MIL-100	25	0.38	0.25		2		2042	4
Cu^+-impregnated Cr-MIL-101	25	2.26	0.05		32		2391	5
Cr-MIL-101	25	1.03	0.21		5		3788	6
Cu^+-impregnated HKUST-1	25	0.59	0.20		10		855	6
HKUST-1	25	0.33	0.22		2		1070	6

$^{\pm}$ Denotes selectivity for a 50:50 mixture calculated using the Ideal Adsorbed solution theory unless otherwise stated
*Denotes ideal selectivity

2. Experimental Section

2.1. Chemicals

All reagents and standards used in this study were of analytical grade and used without further purification. Acetonitrile (MeCN, 99.8%), cobalt(II) nitrate hexahydrate ($Co(NO_3)_2{\cdot}6H_2O$, 98%), copper(II) chloride dihydrate ($CuCl_{2.}2H_2O$, 99.9%), copper(II) nitrate trihydrate ($Cu(NO_3)_2.3H_2O$, 99%), copper(II) oxide (CuO, 99.9%), copper(I) oxide (Cu_2O, 99.9%), 2,5-dihydroxyterephthalic acid (H_2DOBDC, 98%), copper powder (Cu, 99.99%) and nickel(II) acetate tetrahydrate ($Ni(OCOCH_3)2{\cdot}4H_2O$, 98%) were purchased from Sigma-Aldrich. N,N-dimethylformamide (DMF, 99.9%), ethanol (EtOH, 99.8%), methanol (MeOH, 99.8%), nitric acid (HNO_3, 69%), 2-propanol (IPA, 99.6%) and tetrahydrofuran (THF, 99.7%) were purchased from VWR. Copper(I)_chloride (CuCl, 99.9%) and copper(II) formate tetrahydrate ($Cu(HCO_2)_{2.}4H_2O$, 98%) were purchased from Alfa Aesar. Copper(II) nitrate tetrahydrate ($Cu(NO_3)_2.4H_2O$, 99% was purchased from Acros Organics. CO (minimum 99.999% purity), He (minimum 99.999% purity) and N_2 (99.999% purity) were all purchased from BOC.

2.2 Materials synthesis

2.2.1 Ni-MOF-74 synthesis

Ni-MOF-74 was synthesized based on a previously reported experimental procedure[7] which was modified to increase product yield. 0.933 g nickel(II) acetate tetrahydrate ($Ni(OCOCH_3)2{\cdot}4H_2O$), 0.373 g H_2DOBDC (2,5-dihydroxyterephthalic acid), 25 mL THF and 25 mL H_2O were combined in a beaker and sonicated until dissolved. The contents were transferred into a Teflon liner and sealed inside a stainless steel autoclave. The autoclave and its contents were heated in a convection oven for 3 days at 110 °C. After cooling, the contents were recovered and the supernatant was decanted and replaced by DMF. The product was soaked in DMF for 2 days, replaced with fresh DMF 4 times during this duration. The same procedure was repeated with MeOH. The final product was collected after decanting the MeOH and drying under N_2 flow. Sample activation for characterisation and testing was performed at 250 °C.

2.2.2 Co-MOF-74 synthesis

Co-MOF-74 was synthesized based on a previously reported experimental procedure[8] and modified for sample activation and scaled down for the reaction vessel used. 1.07 g cobalt(II) nitrate hexahydrate ($Co(NO_3)_2{\cdot}6H_2O$), 0.357 g H_2DOBDC (2,5-dihydroxyterephthalic acid), 50 mL DMF, 50 mL EtOH and 50 mL H_2O were combined in a beaker and sonicated until dissolved. The contents were transferred to a 250 mL Schott bottle and heated in a convection oven for 66 h at 100 °C. After cooling, the contents were recovered and the supernatant was decanted and replaced by DMF. The product was soaked in DMF for 2 days, replaced with fresh DMF 4 times during this duration. The same procedure was repeated with MeOH. The final product was collected after decanting the MeOH and drying under N_2 flow. Sample activation for characterisation and testing was performed at 250 °C.

2.3 X-ray absorption fine structure (XAFS) analysis

2.3.1 Copper standards

The Cu standard used for the XAFS analysis were: CuO, $Cu(HCO_2)_2.4H_2O$, $Cu(NO_3)_2.4H_2O$, Cu_2O, $CuCl_2{\cdot}2H_2O$, CuCl and Cu.

2.3.2 Sample preparation and activation

The samples, after appropriate dilution in boron nitride, were loaded into an open-ended capillary (ø = 3 mm, Length = 100 mm) and mounted on a catalysis testing station, with the capillary connected to gas lines. During the measurements, the samples were heated from room temperature to 250 °C under He atmosphere, stopping at 80 °C and 160 °C to collect higher quality spectra. After reaching the temperature of 250 °C, the atmosphere was changed to 10% CO in He and measurements were collected continuously until no changes of the Cu-edge XANES spectra could be observed. Samples were then brought down to room temperature and XAFS spectra were collected under a CO and N_2 atmospheres.

2.3.3 Data acquisition

XAFS data were acquired at Cu K edge (8.98 keV) for both samples and Ni K edge (8.33 keV) and Co K edge (7.71 keV), for Cu@Ni-MOF-74 and Cu@Co-MOF-74 respectively. Ni and Co edge measurements were performed in transmission mode using ion chambers, while fluorescence mode, acquired using a 64 elements Ge detector, was required for Cu due to the low Cu amount in the sample. Unfortunately it was not possible to collect Cu-edge XAFS spectra under $CO:N_2$ atmosphere for the Cu@Co-MOF-74 due to experimental issues, but Co-edge

XAFS measurement were obtained. A foil of the same element was collected simultaneously to allow for alignment to uniform energy.

2.3.4 Data analysis

XAFS data processing and analysis was performed using the Athena and Artemis software from the Demeter IFEFIT package.[9] Due to the high signal to noise ratio and short acquisition range, only XANES information could be obtained from the Cu edge data. For Ni and Cu, the resultant EXAFS data have been used to determine changes in structure of the MOF structure. The FEFF6 code was used to construct theoretical EXAFS signals, which included single-scattering contributions from atomic shells through the nearest neighboring atoms in the MOF. The *k*-range used for the fitting went from 3 to 10.634 $Å^{-1}$ and the *r*-range from 1.305 to 3.5 Å. The path degeneracy was kept constant in the fit and the amplitude reduction factor (S_0) was fixed at 0.800. In particular, the path taken in exam are the M-O and M-M for all spectra, and M-C for the sample under CO atmosphere at room temperature.

2.4. Flux response technology (FRT) instrument experimental

2.4.1. FRT schematic

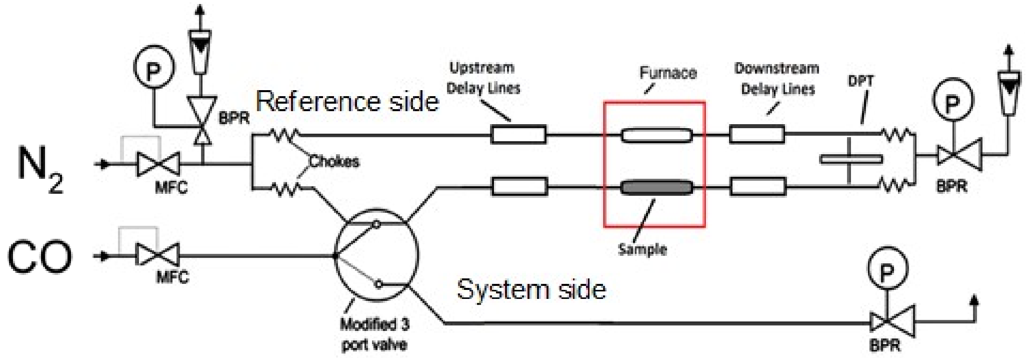

Figure S1. Schematic of flux response technology instrument for dynamic CO adsorption measurements. MFC = Mass-flow controller; BPR = Back-pressure regulator; DPT = Differential pressure transducer.

2.4.2. FRT profile

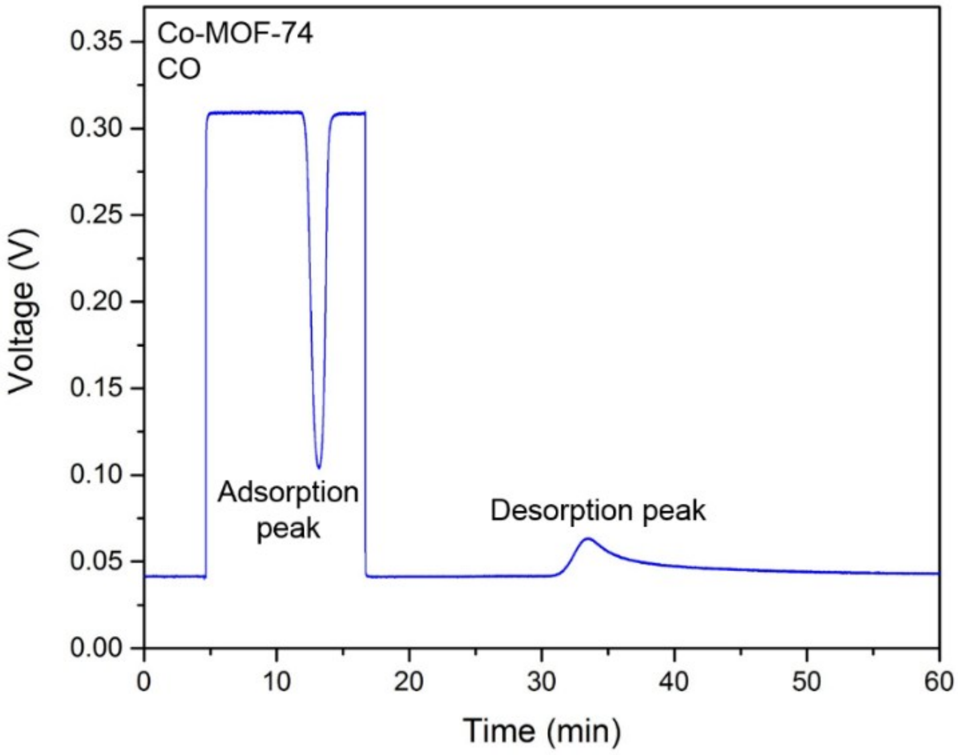

Figure S2. Flux response technology experimental profile of Co-MOF-74 (50:50 *v:v* CO:N_2) at 25 °C and 1 bar pressure.

2.5. Breakthrough adsorption column experimental

2.5.1. Breakthrough adsorption column schematic

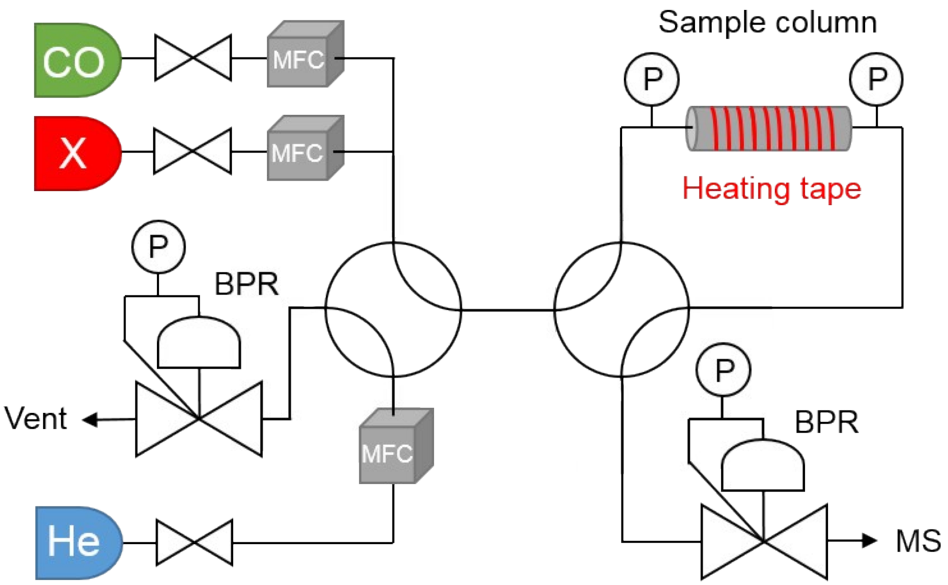

Figure S3. Schematic of breakthrough adsorption column instrument for CO/X separation measurements (X = N_2, CO_2). MFC = Mass-flow controller; BPR = Back-pressure regulator; MS = Mass spectrometer.

2.5.2. Breakthrough column measurement calculations

The adsorbate, CO in the example below, can be substituted with N_2 or CO_2 for its corresponding calculations. The dead volume of the system and total voidage of the adsorbent bed must be accounted for in order to calculate the adsorption capacity. Here (Eq 1), overall material balance of the helium can be expressed as the upstream dead volume, downstream dead volume and the total voidage of the adsorbent bed.

$$\{V_{d,u} + V_{d,d} + V_b[\varepsilon_b + (1 - \varepsilon_b)\varepsilon_p]\}c_t y_{He} = \int_0^{\infty} (\dot{n}_i y_{He} - \dot{n}_o y_{He,o})dt \quad (1)$$

Overall material balance on the adsorbate:

$$\{V_{d,u} + V_{d,d} + V_b[\varepsilon_b + (1 - \varepsilon_b)\varepsilon_p]\}c_t y_X + m_b q_X = \int_0^{\infty} (\dot{n}_i y_{CO} - \dot{n}_o y_{CO,o})dt \quad (2)$$

An approximation of the overall material balance:

$$\dot{n}_o \approx \dot{n}_i \frac{(1 - y_{CO})}{(1 - y_{CO,o})} \quad (3)$$

Combining both equations provides the saturation capacity (*SC*, total adsorption uptake when $y_{CO,\,o} = y_{CO}$ at the detector):

$$SC_{CO} = \frac{\dot{n}_i y_X}{m_b} \int_{t=0}^{t_{at\, y_{CO,o} = y_{CO}}} \frac{(1 - y_{CO})}{(1 - y_{CO,o})} \left(\frac{y_{He,o}}{y_{He}} - \frac{y_{CO,o}}{y_{CO}} \right) dt \quad (4)$$

Breakthrough capacity (*BC*, adsorption uptake when $\frac{y_{CO,\,o}}{y_{CO}} = 0.01$ at the detector) can be calculated using the following:

$$BC_{CO} = \frac{\dot{n}_i y_X}{m_b} \int_{0}^{t_{at \frac{y_{CO,\,o}}{y_{CO}} = 0.01}} \frac{(1 - y_{CO})}{(1 - y_{CO,o})} \left(\frac{y_{He,o}}{y_{He}} - \frac{y_{CO,o}}{y_{CO}} \right) dt \quad (5)$$

Ideal selectivity (IS) at equilibrium can be calculated using the following ($X = N_2, CO_2$):

$$IS = \frac{SC_{CO}}{SC_X} \tag{6}$$

Where

$V_{d,u}$	Dead volume in the upstream pipework (m^3)
$V_{d,d}$	Dead volume in the downstream pipework (m^3)
V_b	Volume of the bed containing the adsorbent (m^3)
ε_b	Total interstitial voidage between the adsorbent particles comprising the bed (-)
ε_p	Voidage inside the adsorbent particles (-)
c_t	Total gas molar concentration (mol m^{-3})
m_b	Mass of adsorbent within the bed (kg)
q_{CO}	Amount of CO adsorbed on the surface of the adsorbent (mol kg^{-1})
y_{He}	Mole fraction of helium in the feed gas (-)
y_{CO}	Mole fraction of CO in the feed gas (-)
y_X	Mole fraction of X in the feed gas (-, $X = N_2, CO_2$)
$y_{He,o}$	Mole fraction of helium detected at the outlet (-)
$y_{CO,o}$	Mole fraction of CO detected at the outlet (-)
$y_{X,o}$	Mole fraction of X detected at the outlet (-, $X = N_2, CO_2$)
$\dot{n}_i$	Molar flow of gas fed into the bed (mol min^{-1})
$\dot{n}_o$	Molar flow of gas out of the bed (mol min^{-1})
t	Time (min)

3. Results

3.1 Materials characterisation

Table S2. Metal loading quantities and textural parameters derived from the elemental analysis (XPS and ICP-MS) and N_2 sorption isotherms at -196 °C for the synthesized and Cu impregnated MOFs. The MOFs were loaded with equimolar solutions of $Cu(II)Cl_2$:$Cu(II)(HCOO)_2$. (S_{BET} = BET surface area, V_{tot} = total pore volume, V_{mic} = micropore volume and V_{mes} = mesopore volume)

**Materials**	**Bulk Cu loading*** (Cu:M % weight)	**Surface Cu loading**† (Cu:M % weight)	**Metal density±** (mmol $_{metal}$ $g^{-1}{}_{adsorbent}$)	**CO uptake normalised for Cu loading** (mmol $_{CO\ adsorbed}$ $mmol^{-1}{}_{Cu}$)	$\mathbf{S}_{BET}$ (m^2 g^{-1})	$\mathbf{V}_{tot}$ (cm^3 g^{-1})	$\mathbf{V}_{mic}$ (cm^3 g^{-1})	$\mathbf{V}_{mes}$ (cm^3 g^{-1})
Ni-MOF-74	-	-	3.97	-	1427	0.58	0.53	0.05
2-Cu@ Ni-MOF-74	1.77	18.95	4.00	0.33	1259	0.49	0.48	0.01
4-Cu@ Ni-MOF-74	3.50	31.86	4.05	3.08	1047	0.43	0.40	0.03
7-Cu@ Ni-MOF-74	7.07	35.73	4.12	1.40	1036	0.44	0.40	0.04
Co-MOF-74	-	-	3.97	-	1520	0.59	0.58	0.01
2-Cu@ Co-MOF-74	2.42	-	4.02	2.4	1204	0.47	0.46	0.01

* Calculated by using the ratio of μg L^{-1} concentrations of Cu and Ni measured by inductively coupled plasma mass spectrometry.

† Calculated by using the ratio of atomic percentage of Cu and Ni measured by X-ray photoelectron spectroscopy.

± The bulk Cu loading, desolvated adsorbent's general formula and crystallographic density was used to calculate the metal density (mol of coordinatively unsaturated metal sites per gram of adsorbent).

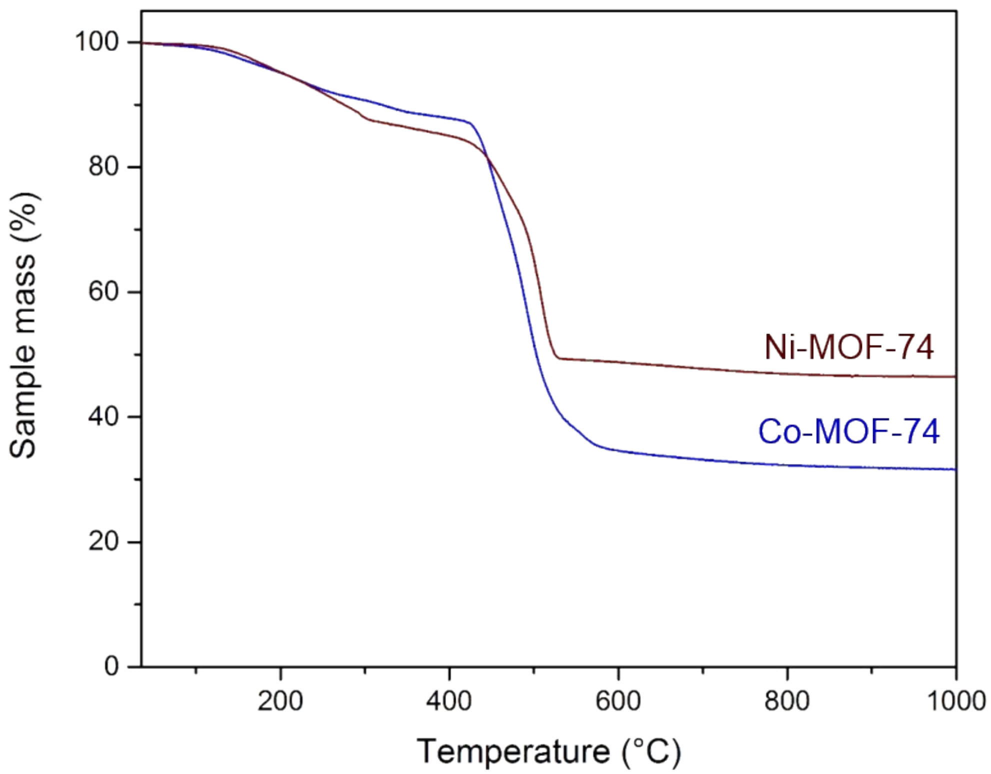

Figure S4. Thermogravimetric curve of Ni-MOF-74 and Co-MOF-74 under N_2 atmosphere.

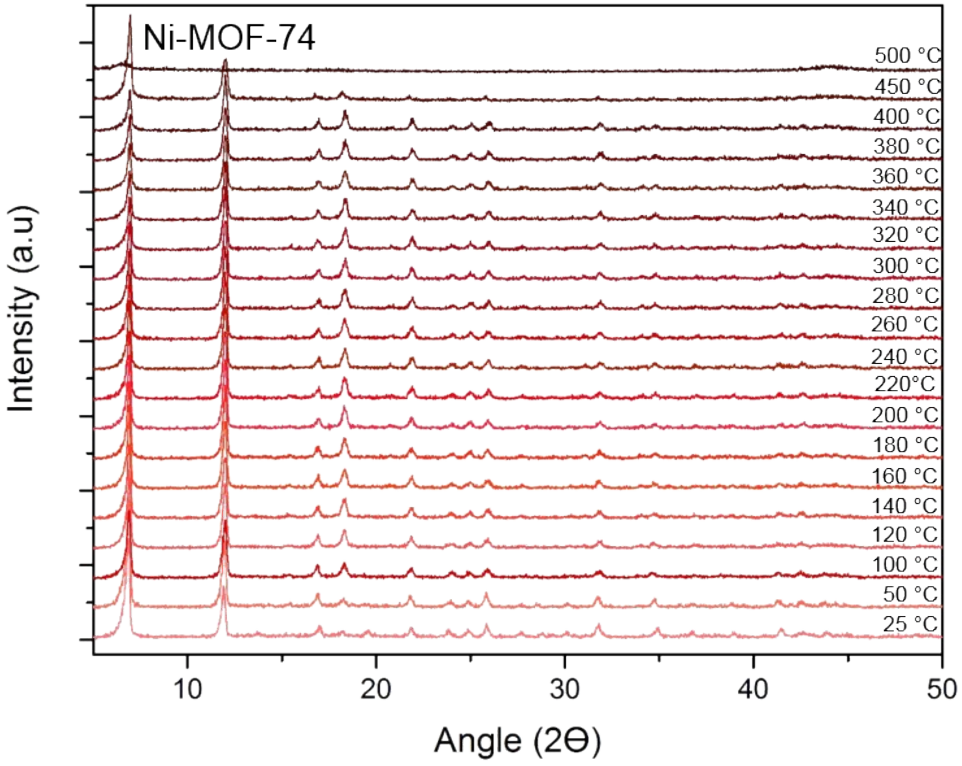

Figure S5. Variable-temperature X-ray diffraction patterns of Ni-MOF-74 from room temperature to 500 °C under vacuum.

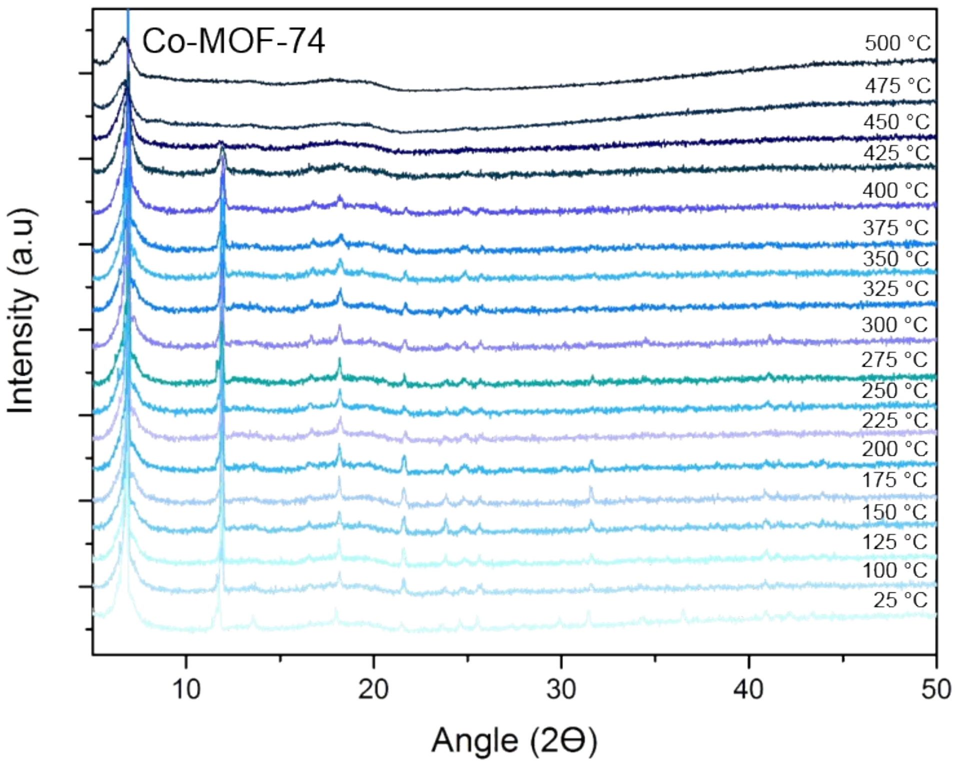

Figure S6. Variable-temperature X-ray diffraction patterns of Co-MOF-74 from room temperature to 500 °C under vacuum.

3.2 Elemental analysis

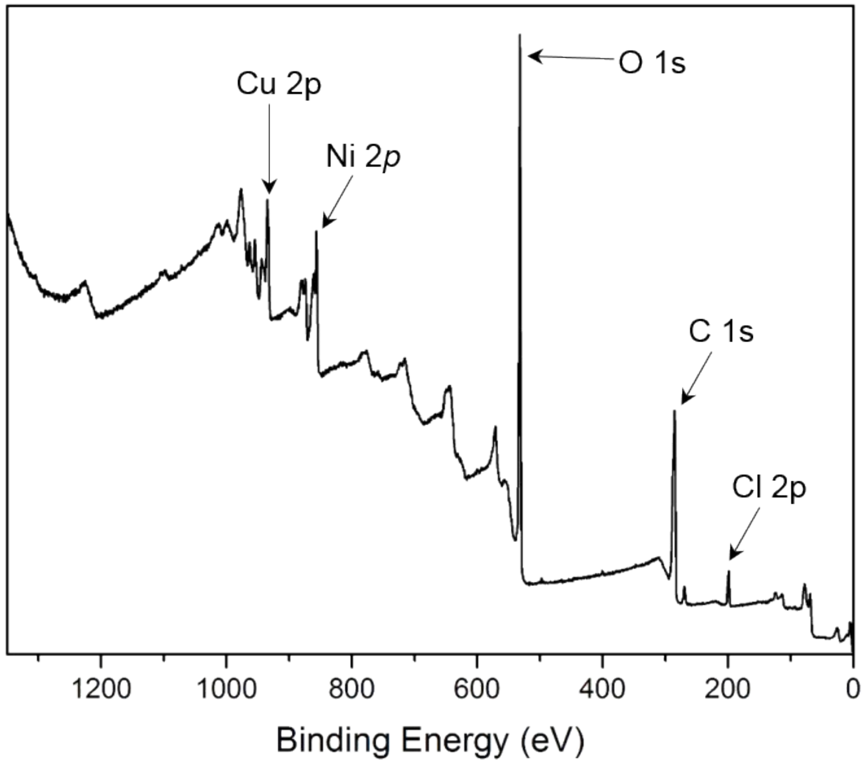

Figure S7. Full XPS spectrum of 4-Cu@Ni-MOF-74.

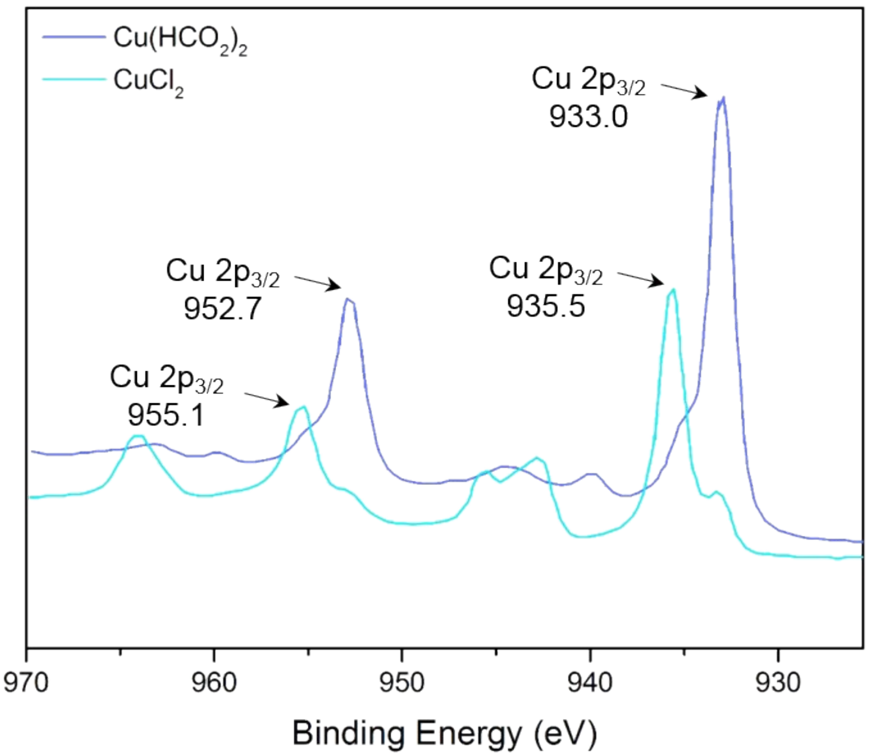

Figure S8. Cu 2*p* spectra of the Cu impregnation salt precursors $Cu(HCO_2)_2$ and $CuCl_2$. The responsible Cu peaks and energy levels are shown on the figure.

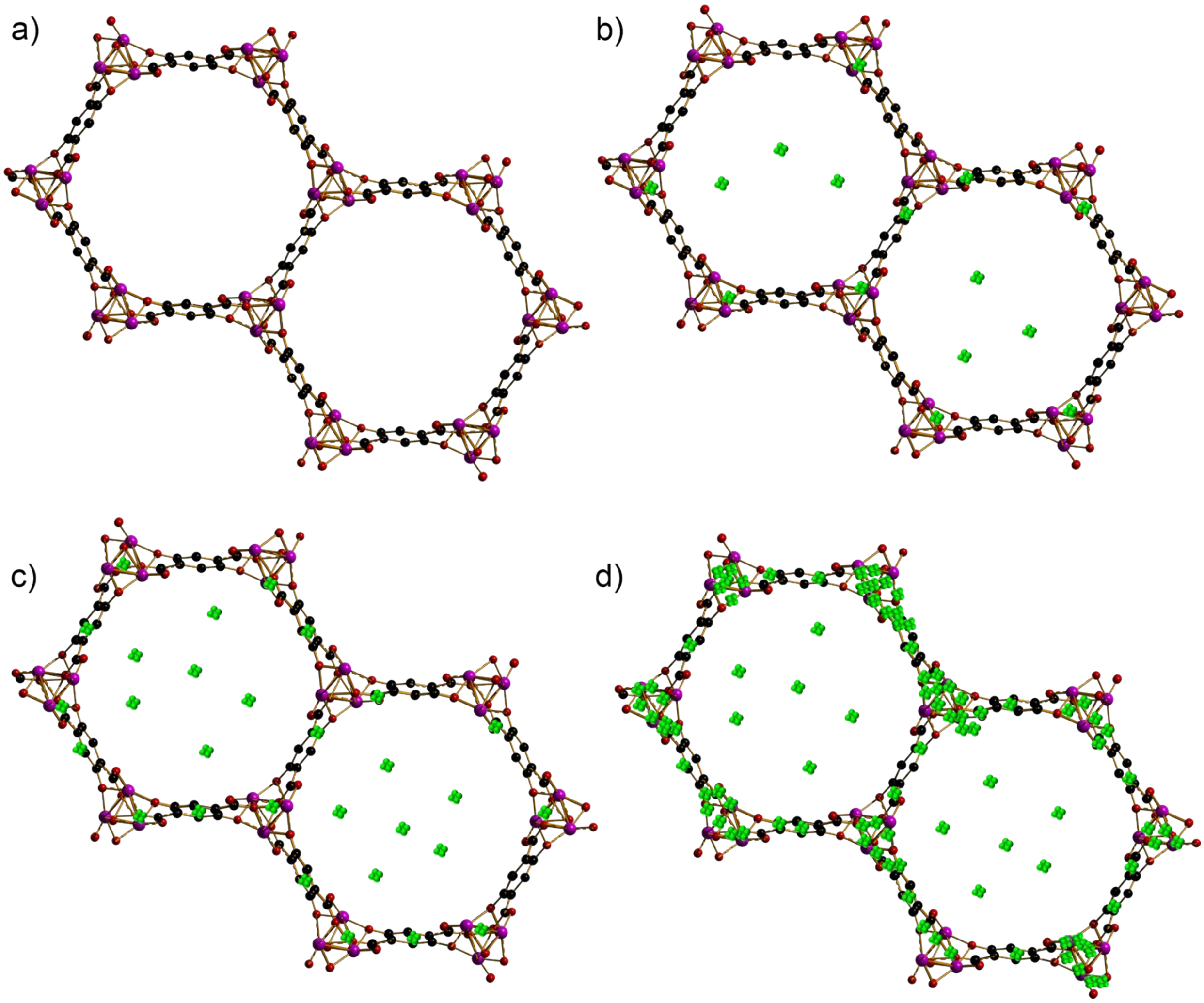

Figure S9. Structures of Cu impregnated Ni-MOF-74 with increased Cu loading: a) Ni-MOF-74, b) 2-Cu@Ni-MOF-74, c) 4-Cu@Ni-MOF-74 and d) 7-Cu@Ni-MOF-74. Black: carbon atoms. Red: oxygen atoms. Purple: nickel atoms. Green: copper clusters (Not to scale).

3.3 In-situ materials characterization

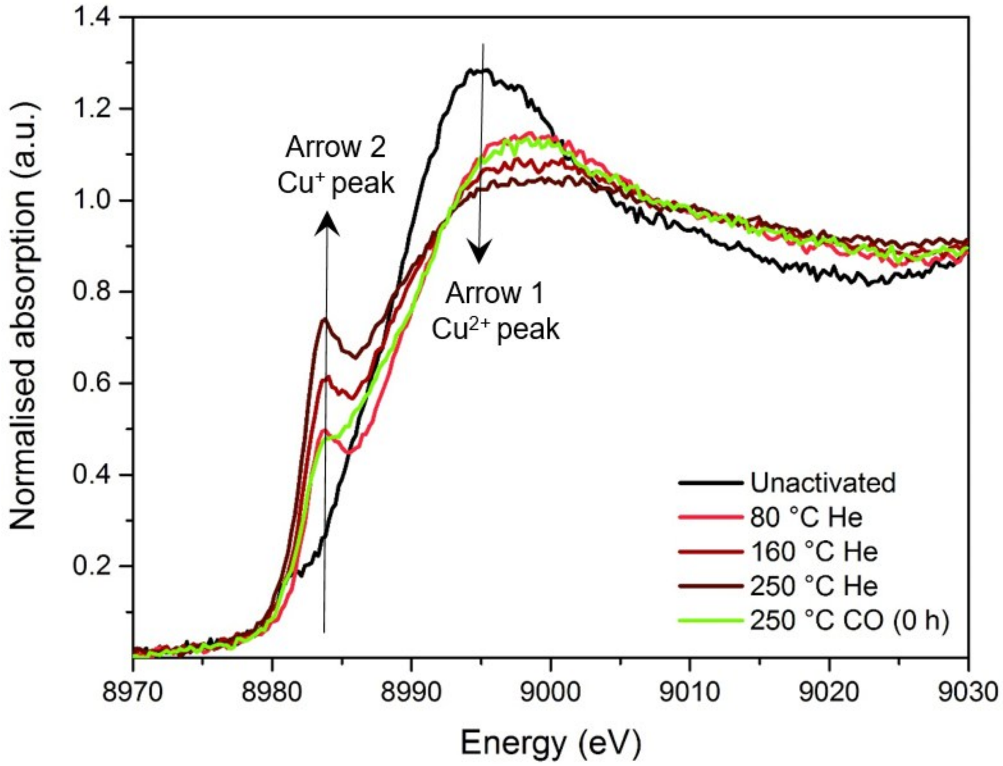

Figure S10. Cu-edge XANES spectra during the initial activation procedure of 4-Cu@Ni-MOF-74 using He and switching to CO. Indicated by arrows are the features for Cu^{2+} (Arrow 1) and Cu^{+} (Arrow 2) peaks.

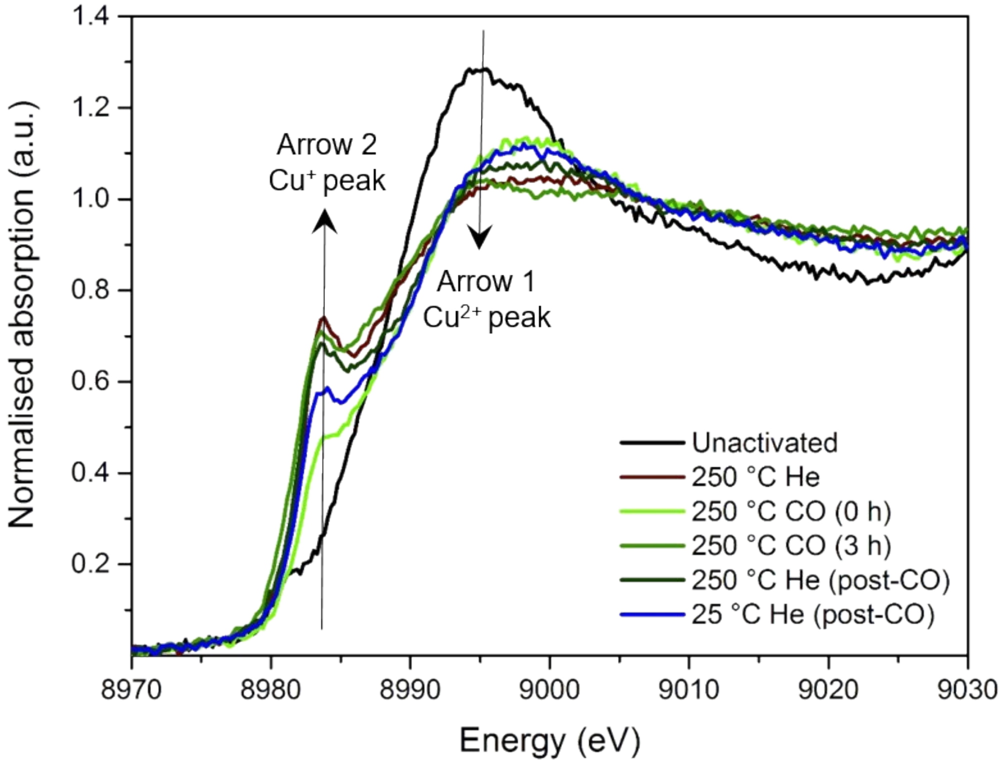

Figure S11. Cu-edge XANES spectra during the activation procedure of 4-Cu@Ni-MOF-74 using CO for Cu reduction. Indicated by arrows are the features for Cu^{2+} (Arrow 1) and Cu^{+} (Arrow 2) peaks.

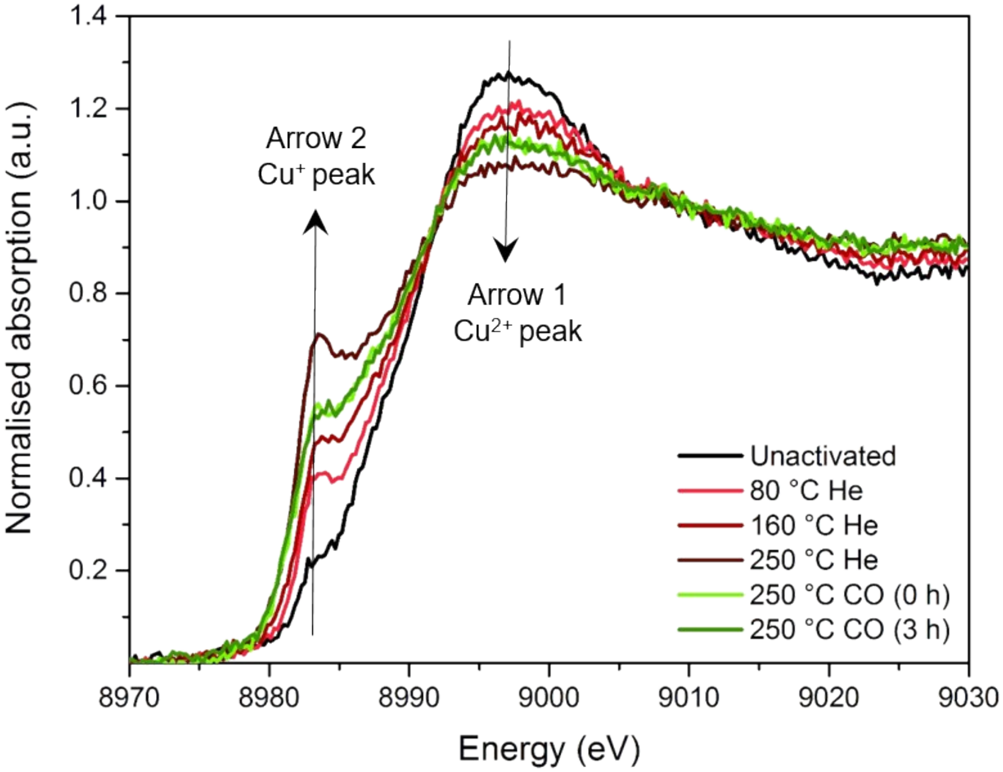

Figure S12. Cu-edge XANES spectra during the MOF activation and Cu reduction procedure of 2-Cu@Co-MOF-74. Indicated by arrows are the features for Cu^{2+} (Arrow 1) and Cu^{+} (Arrow 2) peaks.

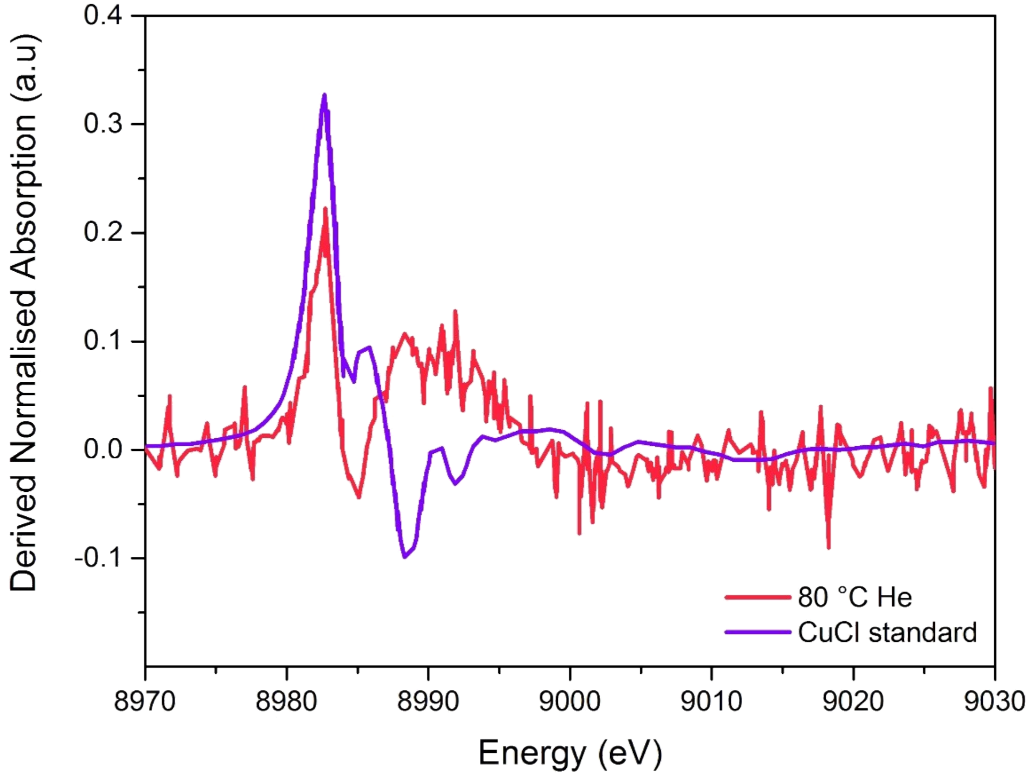

Figure S13. First derivative of the Cu-Edge XANES spectra of CuCl and 4-Cu@Ni-MOF-74 at 80 °C under He atmosphere.

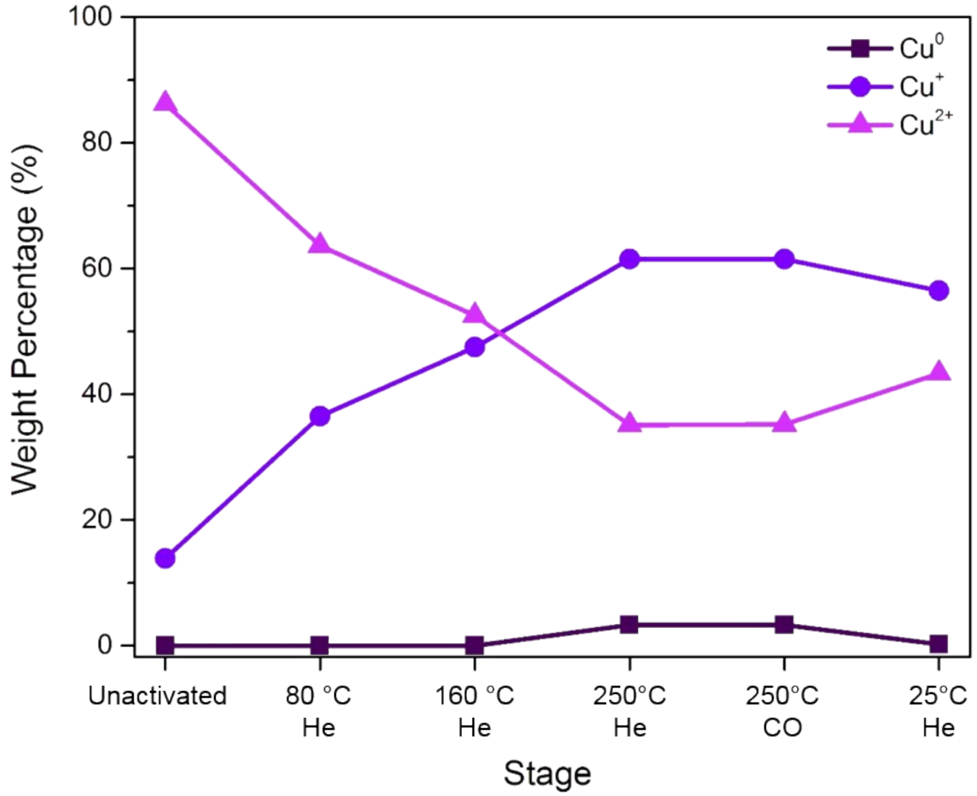

Figure S14. Linear Combination Fit analysis of Cu species in 2-Cu@Co-MOF-74 during the activation and reduction procedure.

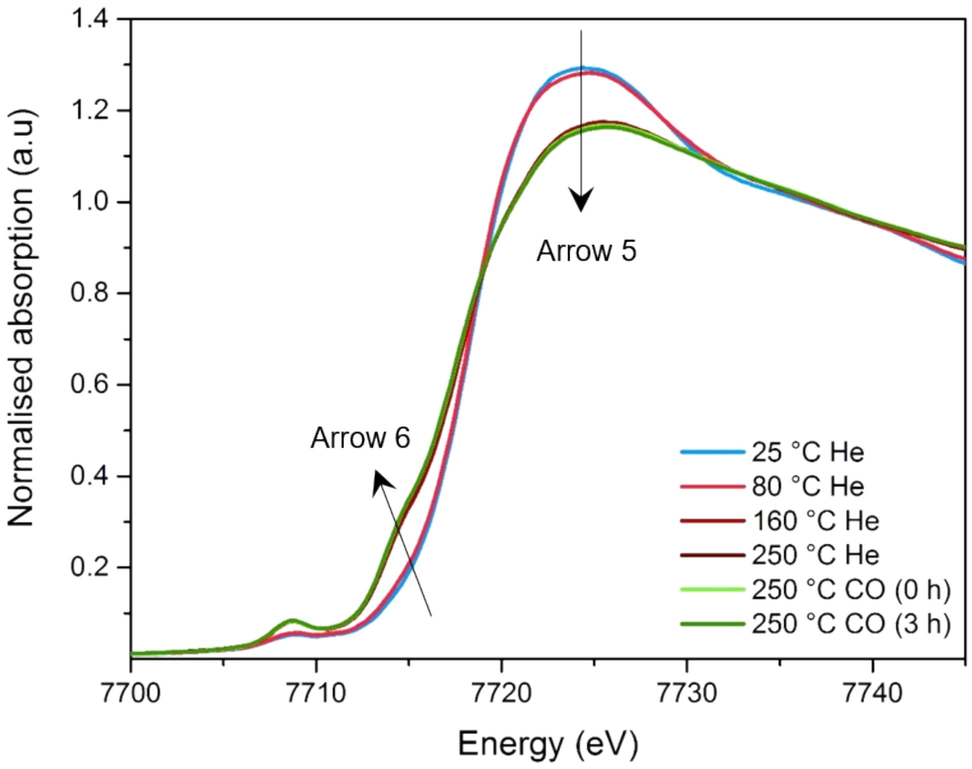

Figure S15. Co-edge XANES spectra of 2-Cu@Co-MOF-74 during the MOF activation and Cu reduction procedure. Indicated by arrows are the features for the white line shift (Arrow 5) and 1*s*-4p_z and 1*s*-3*d* transition (Arrow 6).

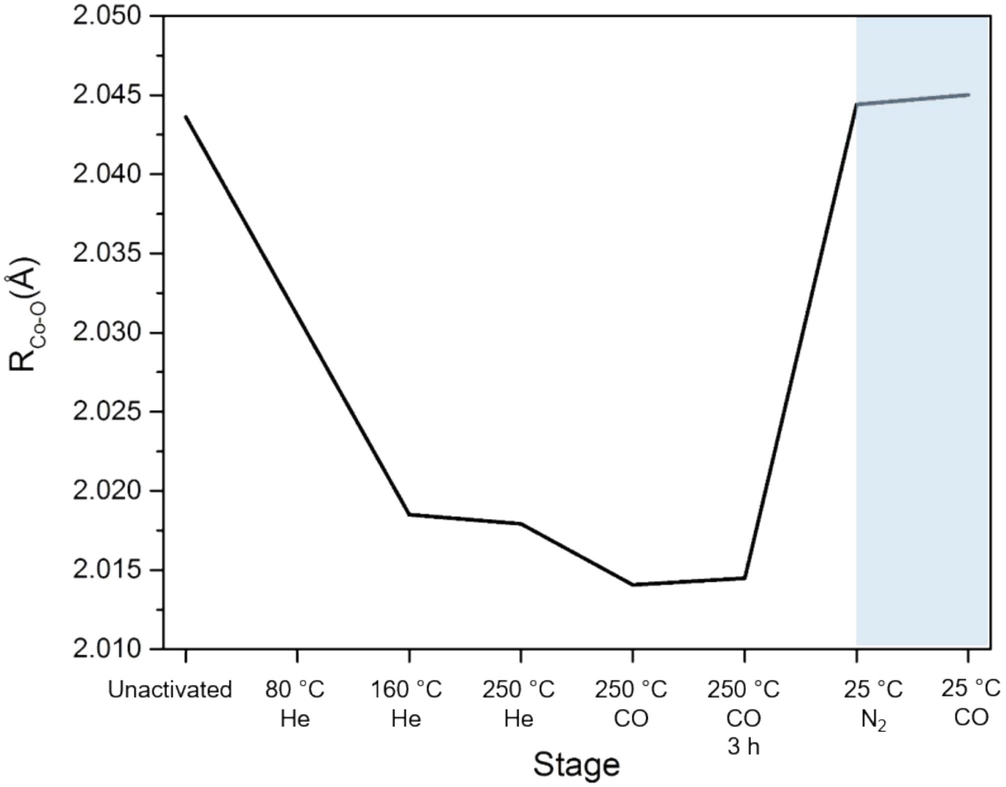

Figure S16. Co-O bond of distance 2-Cu@Co-MOF-74 as obtained by the EXAFS fit of the system at different temperature and atmosphere during the activation and reduction procedure and analysis (shaded).

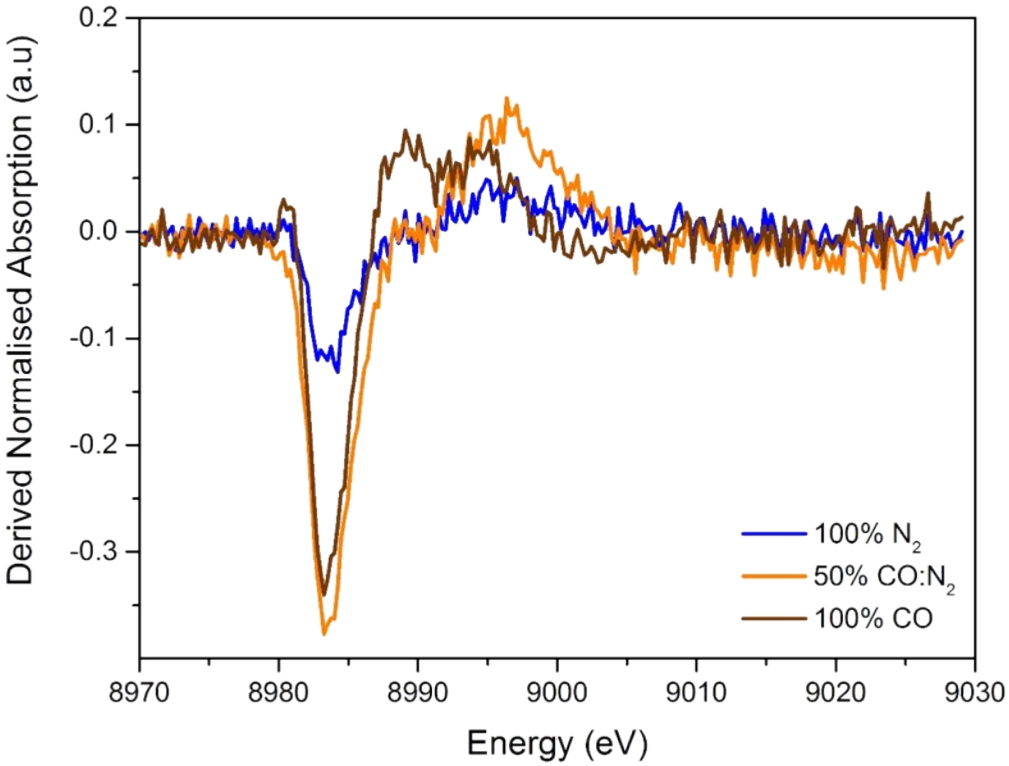

Figure S17. Cu edge Δμ-XANES spectra 4-Cu@Ni-MOF-74 at 25 °C under N_2 and CO atmosphere after activation.

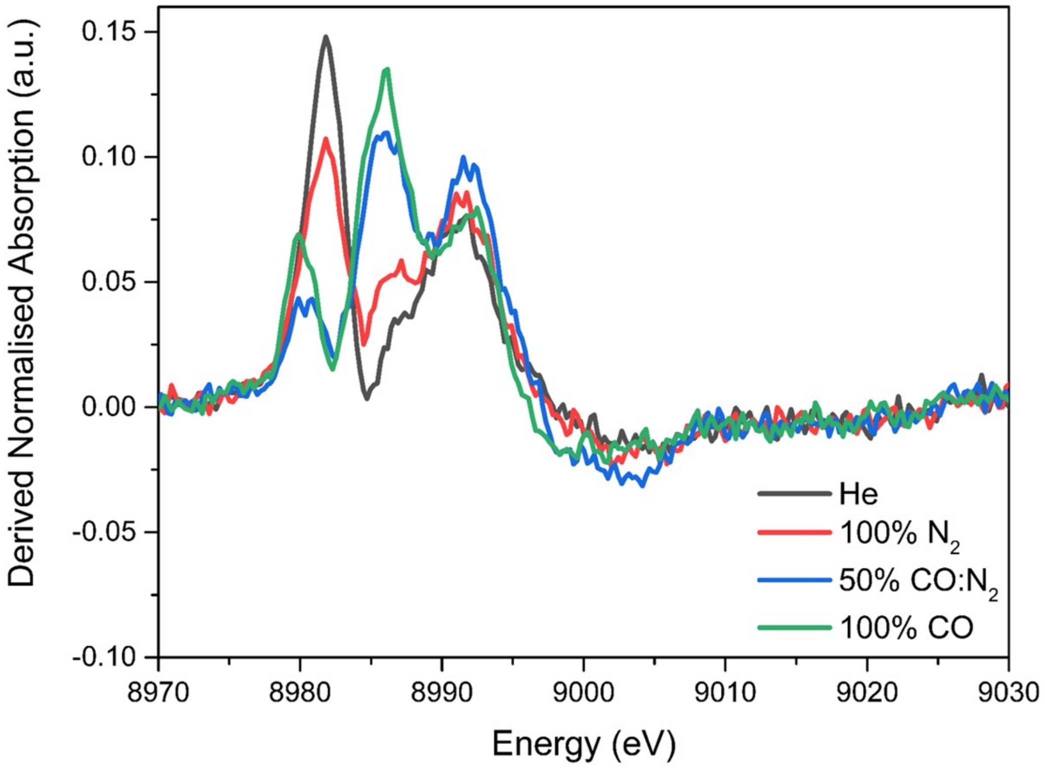

Figure S18. Cu edge Δμ-XANES spectra 2-Cu@COo-MOF-74 at 25 °C under He, N_2 and CO atmosphere after activation.

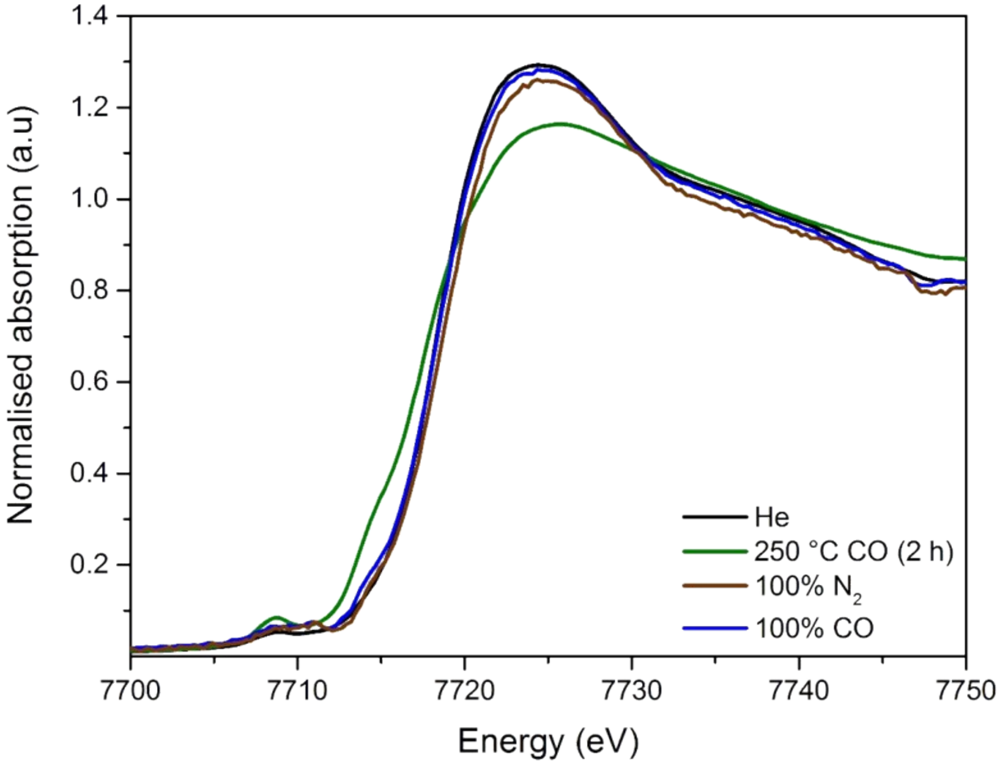

Figure S19. Co-edge XANES spectra of 2-Cu@Co-MOF-74 at 25 °C under He atmosphere before activation and under N_2 and CO atmosphere after activation. The spectra during activation in CO atmosphere at 250 °C is shown in green.

1. Y.-X. Li, Y.-N. Ji, M.-M. Jin, S.-C. Qi, S.-S. Li, D.-M. Xue, M. B. Yue, X.-Q. Liu and L.-B. Sun, *ACS Applied Materials & Interfaces*, 2018, **10**, 40044-40053.
2. A.-R. Kim, T.-U. Yoon, S.-I. Kim, K. Cho, S.-S. Han and Y.-S. Bae, *Chemical Engineering Journal*, 2018, **348**, 135-142.
3. T. K. Vo, Y.-S. Bae, B.-J. Chang, S.-Y. Moon, J.-H. Kim and J. Kim, *Microporous and Mesoporous Materials*, 2019, **274**, 17-24.
4. J. Peng, S. Xian, J. Xiao, Y. Huang, Q. Xia, H. Wang and Z. Li, *Chemical Engineering Journal*, 2015, **270**, 282-289.
5. Y. Wang, C. Li, F. Meng, S. Lv, J. Guo, X. Liu, C. Wang and Z. Ma, *Frontiers of Chemical Science and Engineering*, 2014, **8**, 340-345.
6. Y. Yin, Z.-H. Wen, X.-Q. Liu, L. Shi and A.-H. Yuan, *Journal of Porous Materials*, 2018, 1-7.
7. P. D. C. Dietzel, B. Panella, M. Hirscher, R. Blom and H. Fjellvåg, *Chemical Communications*, 2006, DOI: 10.1039/B515434K, 959-961.
8. T. Grant Glover, G. W. Peterson, B. J. Schindler, D. Britt and O. Yaghi, *Chemical Engineering Science*, 2011, **66**, 163-170.
9. B. Ravel and M. Newville, *Journal of Synchrotron Radiation*, 2005, **12**, 537-541.
